# A novel CD4+ CTL subtype characterized by chemotaxis and inflammation is involved in the pathogenesis of Graves’ orbitopathy

**DOI:** 10.1038/s41423-020-00615-2

**Published:** 2021-01-29

**Authors:** Yue Wang, Ziyi Chen, Tingjie Wang, Hui Guo, Yufeng Liu, Ningxin Dang, Shiqian Hu, Liping Wu, Chengsheng Zhang, Kai Ye, Bingyin Shi

**Affiliations:** 1 Department of Endocrinology, the First Affiliated Hospital of Xi’an Jiaotong University, Xi’an, China; 2 MOE Key Lab for Intelligent Networks & Networks Security, School of Electronic and Information Engineering, Xi’an Jiaotong University, Xi’an, China; 3 Genome Institute, the First Affiliated Hospital of Xi’an Jiaotong University, Xi’an, China; 4 Precision Medicine Center, the First Affiliated Hospital of Xi’an Jiaotong University, Xi’an, China; 5 BioBank, the First Affiliated Hospital of Xi’an Jiaotong University, Xi’an, China; 6 The Jackson Laboratory for Genomic Medicine, Farmington, CT 06032 USA; 7 The School of Life Science and Technology, Xi’an Jiaotong University, Xi’an, China

**Keywords:** Graves’ orbitopathy, single-cell RNA sequencing, CD4+ cytotoxic T lymphocytes, Autoimmunity, Lymphocyte differentiation, CD4-positive T cells

## Abstract

Graves’ orbitopathy (GO), the most severe manifestation of Graves’ hyperthyroidism (GH), is an autoimmune-mediated inflammatory disorder, and treatments often exhibit a low efficacy. CD4+ T cells have been reported to play vital roles in GO progression. To explore the pathogenic CD4+ T cell types that drive GO progression, we applied single-cell RNA sequencing (scRNA-Seq), T cell receptor sequencing (TCR-Seq), flow cytometry, immunofluorescence and mixed lymphocyte reaction (MLR) assays to evaluate CD4+ T cells from GO and GH patients. scRNA-Seq revealed the novel GO-specific cell type CD4+ cytotoxic T lymphocytes (CTLs), which are characterized by chemotactic and inflammatory features. The clonal expansion of this CD4+ CTL population, as demonstrated by TCR-Seq, along with their strong cytotoxic response to autoantigens, localization in orbital sites, and potential relationship with disease relapse provide strong evidence for the pathogenic roles of GZMB and IFN-γ-secreting CD4+ CTLs in GO. Therefore, cytotoxic pathways may become potential therapeutic targets for GO.

## Introduction

Graves’ hyperthyroidism (GH) is an organ-specific autoimmune disease with a lifetime risk of 3% in women and 0.5% in men.^1^ Graves’ orbitopathy (GO), which is characterized by disfiguring and dysfunctional manifestations of eyelid retraction, proptosis or optic neuropathy, occurs in 20–50% of GH patients.^2^ GO involves autoimmune-mediated inflammation of the orbit characterized by fibroblast activation, adipogenesis and enlargement of the extraocular muscles. However, the precise pathogenesis of GO remains poorly understood.^3^ Approximately 20–30% of patients have no response to glucocorticoids, the mainstay treatment for GO, and 10–20% relapse after treatment withdrawal.^2^ Based on current knowledge of GO pathogenesis, novel clinical trials for various treatments, including mycophenolate^4,5^ and azathioprine^6^ targeting the overactivation and proliferation of lymphocytes and the IGF-IR inhibitors teprotumumab,^7,8^ tocilizumab^9^ and selenium,^10^ have been conducted. Therefore, addressing the pathogenesis of GO has always been recognized as imperative for the identification of new therapeutic targets.

CD4+ T cells are the most abundant lymphocytes infiltrating GO orbital tissues and have been reported to initiate and perpetuate orbital inflammation in GO.^11,12^ Orbital fibroblasts, as the target cells in GO, are specifically activated by the T cell receptor (TCR) on antigen-specific CD4+ T cells^13^ and perpetually stimulated by T cells expressing cytokines and chemokines, such as interferon-γ (IFN-γ), tumor necrosis factor and C–X–C motif ligands.^14^ Furthermore, upon exposure to their cognate antigen, CD4+ T cells undergo differentiation and can be classified as T helper (Th)1 cells, Th2 cells, regulatory T cells, Th17 cells and newly reported CD4+ cytotoxic T lymphocytes (CTLs) on the basis of functional properties.^15,16^ Previous studies have shown that the balance between Th1 and Th2 cells is involved in the progression of GO, although some findings have contradicted these results.^17,18^ The pathogenicity of Th17 cells in the initiation and progression of GO was also confirmed according to antigen-presenting functions and proinflammatory cytokines.^19,20^ A recent GO study indicated that the immune microenvironment in the orbit is composed of natural killer (NK) cells, IFN-γ-producing and RORγt+TBX21+ T cells and CD34+ orbital fibroblasts.^21^

CD4+ CTLs were initially identified in chronic viral infections or vaccinations against certain viral infections.^22–24^ They have also been reported to undergo expansion in the peripheral blood or diseased tissue under inflammatory conditions, such as antitumor immune responses or autoimmune disorders.^25–29^ Systemic sclerosis, an autoimmune fibrotic disease, was reported to involve CD4+ CTL-induced apoptotic death of endothelial and other cells.^27^ Mass cytometry and flow cytometry analyses of rheumatoid arthritis patients revealed an expanded population of CD27-HLA-DR+ cells that expressed Th1- and cytotoxicity-associated features and produced abundant IFN-γ and granzyme A proteins.^28^ Studies of IgG4-related disease have also shown that CD4+ effector/memory T cells with a cytotoxic phenotype are expanded in inflamed tissue sites and secrete the profibrotic cytokines IFN-γ, IL-1β and TGF-β1.^30,31^ Granzymes stored in CTLs directly influence inflammation by releasing proinflammatory cytokines, including IL-6, tumor necrosis factor (TNF) α and IL-1β, which are affected by GZMA, GZMB, GZMK and GZMM.^32–34^ Granzymes can also degrade several extracellular matrix components, contributing to inflammation and mediating tissue remodeling.^35,36^ Thus, further characterization of CD4+ T cell subsets may better decipher the immune mechanisms of GO progression.

Given the importance of CD4+ T cells in the pathogenesis of GO, we undertook unbiased single-cell RNA sequencing (scRNA-Seq) and identified a novel CD4+ CTL subtype with chemotactic and inflammatory properties; the subset comprised GO-specific, clonally expanded, granzyme-releasing cells and was present as a large fraction of the total CD4+ T cell population within orbital tissues. Moreover, increased levels of cytotoxicity and chemotaxis in CD4+ CTLs were probably correlated with GO relapse. Our findings suggest that this novel proinflammatory CD4+ CTL subtype contributes to the pathogenesis of GO and is worth exploring as a new treatment target in the future.

## Materials and methods

### Study design

This study was approved by the Ethics Committee of the First Affiliated Hospital of Xi’an Jiaotong University (XJTU1AF2016LSK-35), and the clinical trial registration number is ChiCTR-IPR-16009305. Informed consent was acquired from all patients before they were screened or included in the study. No statistical methods were used to predetermine the sample size.

To evaluate pathogenic CD4+ T cell subsets and explore the treatment effect on GO, we performed a single-cell sequencing study of six GO and three GH patients (Supplementary Tables 1–2). Blood samples were obtained from treatment-naïve GH patients (newly diagnosed GH patients not yet given any treatment) and from GO patients before and after therapy. All diagnoses were based on clinical symptoms, serum indexes of thyroxine, anti-thyroid stimulating hormone receptor autoantibodies (TRAbs), thyroid ultrasound images and computed tomography scans of the orbit. The details of the inclusion and exclusion criteria are provided in Supplementary Table 1. Four newly diagnosed GO patients (A03, A04, A07 and B01) received high-dose intravenous methylprednisolone (IVMP) therapy, which consisted of 0.5 g administered every other day for a total of 3 times and repeated at intervals of 20 days for a total of 3 cycles. A total of 10 healthy patients without thyroid-related disorders who had no autoimmune disease history or infection within the last 3 months were recruited for the control group and were matched by age and sex.

### Flow cytometry analysis and sorting

Peripheral blood mononuclear cells (PBMCs) were isolated from 10 ml fresh peripheral blood samples by Ficoll-Paque (GE Healthcare, USA) density gradient centrifugation. PBMCs were suspended in flow cytometry staining buffer (eBioscience, San Diego, CA, USA) at a final concentration of 10^7^ cells/ml. After incubation with Fc-blocking antibodies (BD Biosciences), cells were labeled with antibodies specific for surface markers at 4 °C for 30 min. For intracellular staining, including staining for transcription factors (Foxp3) and cytotoxic molecules (granzyme A, granzyme B, granulysin and perforin 1), cells were fixed and permeabilized with a transcription factor buffer set (BD Biosciences) according to the manufacturer’s guidelines and then stained for intracellular proteins at 4 °C for 45 min. The results were read on a Canto II instrument (BD Biosciences) and analyzed using FlowJo software version 10.0.7 (Tree Star). The following monoclonal antibodies were used: anti-CD4 (clone RPA-T4), anti-CCR6 (clone 11A9), anti-CCR4 (clone 1G1), anti-CXCR3 (clone 1C6), anti-CD45RA (clone Hi100), anti-CCR7 (clone 150503), anti-ICOS (clone DX29), and anti-PD-1 (clone EH12.1) from BD Biosciences; anti-CD25 (clone BC96), anti-CXCR5 (clone MU5UBEE), anti-Foxp3 (clone PCH101), and anti-CD45RO (clone UCHL1) from eBioscience; and anti-granzyme A (clone CB9), anti-granzyme B (clone QA16A02), anti-killer cell lectin-like G1 (KLRG1; clone SA231A2), anti-granulysin (clone DH2), anti-FGFBP2 (clone TDA3), and anti-Perforin 1 (clone B-D48) from BioLegend.

For cell sorting, PBMCs were prepared as described above. After labeling with anti-CD3 and anti-CD4 antibodies (BD Biosciences, San Jose, CA, USA) for 30 min at 4 °C in FACS buffer (2% FBS in Dulbecco’s PBS), viable CD3+ CD4+ T cells were sorted into PBS+ 0.04% BSA by fluorescence-activated cell sorting (FACS; FACSAria, BD) and retained on ice. Sorted cells were confirmed to be >90–95% pure before RNA extraction or scRNA-Seq. A MACS bead system (Miltenyi, Bergisch, Germany) was used to sort CD4+ KLRG1+ and naïve CD4+ T cells according to the manufacturer’s guidelines. Sorted cells were confirmed to be >85–95% pure before RNA extraction.

### scRNA-Seq

FACS-sorted CD4+ T cells were suspended in ice-cold PBS+ 0.04% BSA and then counted and assessed for viability with Trypan blue staining. Cells were resuspended at a concentration of 5 × 10^5^–1 × 10^6^ cells/ml with a final viability >80%. A single-cell library was prepared following the protocol of the v2 Reagent Kit from 10× Genomics^37^ (10× Genomics, CA, USA) aiming for an estimated 8000 cells per library. Briefly, cell suspensions were processed in the chip (10× Genomics) along with reverse transcription (RT) master mix and single-cell 3’ gel beads. After RT, barcoded cDNA was purified, followed by PCR amplification. Then, the cDNA was fragmented and double size selected with SPRI beads. Libraries were sequenced on an Illumina NovaSeq platform using paired-end sequencing (PE150 bp).

### Single-cell sequencing data processing

The Cell Ranger software pipeline (version 2.1.1) provided by 10x Genomics was used to demultiplex cellular barcodes and map reads to a genome (GRch38) and transcriptome (STAR aligner), producing a matrix of gene counts versus cells. We processed the unique molecular identifier (UMI) count matrix using the R package Seurat (version 2.3.4). As a quality-control (QC) step, we filtered out genes annotated as ribosomal genes or those found in less than three cells and removed cells with fewer than 100 nonzero count genes or with total UMI counts fewer than 1500. To remove likely multiplet captures, which is a major concern in microdroplet-based experiments, we calculated and excluded cells with a transcript count greater than 3 standard deviations away from the mean. We further discarded low-quality cells, in which more than 10% of the counts belonged to mitochondrial genes. After applying these QC criteria, 85,265 single cells and 18,474 genes were included in downstream analyses.

### Unsupervised clustering and determination of the major cell types

To identify pathogenic CD4+ T cell subsets and explore the treatment effects on CD4+ T cell types, we merged samples from treatment-naïve GH (*n* = 3) and GO (*n* = 6) patients or from GO patients before (*n* = 4) and after (*n* = 4) treatment. Library size normalization was performed in each group by Seurat (2.3.4) on the filtered matrix to obtain normalized counts. After normalization, we used Combat to remove batch effects from each group. Then, we applied the mean-dependent trend method in the Scran package (1.10.1) to identify the highly variable genes. The significant genes (false discovery rate (FDR) ≤ 1e−3) were selected^38^ for principal component analysis (PCA) to reveal biologically meaningful variation. To visualize clusters in two dimensions, graph-based cluster detection and the t-SNE algorithm were applied to the top five principal components. The number of components used was determined based on the JackStraw function. We defined the cell types for each cluster with the following two steps.^39^ First, differential expression among clusters was calculated using a likelihood-ratio test for single-cell gene expression implemented in Seurat (2.3.4) at a familywise error rate of 5%. The differentially expressed genes (DEGs) were ranked by their *P* values from smallest to largest. The top-ranking genes for each cluster are listed as cell type markers. The full lists of signature DEGs used for distinguishing clusters are shown in Supplementary File 1. The top 20 significant genes for each cluster were chosen to name the clusters. Second, based on prior knowledge, a set of canonical marker genes linked to known cell types was analyzed to measure the expression levels. The canonical marker genes among the DEGs in a given cluster indicated the functional status.

### Gene set enrichment analysis (GSEA)

To further explore the differences between GO and GH within six CD4+ T cell types, we reanalyzed cells and pathways separately in each cell type. GSEA^40^ was performed using the R program (package fgsea) (http://bioconductor.org/packages/release/bioc/html/fgsea.html). The signal-to noise ratio between GO and GH for certain cell types (log2-fold change (FC)) was used for gene ranking. Two reference gene sets, namely, Gene Ontology (GO) terms from MSigDB and 50 hallmark pathways described in MSigDB, were examined.

### TCRβ CDR3 sequencing

MACS bead system-sorted CD4+ KLRG1+ and naïve CD4+ T cells were first suspended in ice-cold PBS+ 0.04% BSA and then counted and assessed for viability with Trypan blue staining. TCR sequencing^41^ was used to construct a library as follows: total RNA was isolated using TRIzol reagent (Invitrogen, USA), and sample QC was performed with an Agilent 2100 Bioanalyzer. The isolated RNA was converted into cDNA (RevertAid First Strand cDNA Synthesis Kit; Fermentas) with a constant region-specific primer. A multiplex PCR system was applied to amplify the CDR3 region of rearranged TCRB loci. A set of forward primers, each specific to one or a set of functional TCR V β segments, and a reverse primer specific to the constant region of TCRB were used to generate amplicons that covered the entire CDR3 region. After size selection and purification, paired-end sequencing of samples was performed using the Illumina HiSeq 2000 platform.

### Degranulation assay and mixed lymphocyte reactions (MLRs)

For the degranulation assay, CD4+ T cells from GO patients were stimulated with 10 μg/mL anti-human CD3 antibody (clone OKT3, BioLegend) for 6 h and incubated with anti-CD4, anti-KLRG1 and anti-CD107a antibodies for surface staining, followed by permeabilization and intracellular staining for granzyme B as described above.

Thyroid-stimulating hormone receptor (TSHR) serves as the primary autoantigen in GO and GH.^42^ A recombinant human TSHR289 protein with a 6× His tag at the C-terminus secreted by Chinese hamster ovary (CHO) cells was purified from culture supernatants by affinity chromatography and dialyzed against 10 mM Tris (pH 7.4) and 50 mM NaCl. The purity of the TSHR289 protein was verified by SDS-PAGE and HPLC-SEC (~97%). For the MLR assay, CD4+ T cells and PBMCs (CD4−, incubated with 25 μg/mL mitomycin (Selleck) for 30 min at 37 °C, 5% CO2) from GO patients were cocultured at a 1:3 ratio with 25 μg/mL TSHR289 protein, blank (negative control) or 10 μg/mL anti-human CD3 antibody (positive control) for 24 h or 48 h. For the CD25 and CD134 assays, 25 μg/mL ovalbumin (OVA, Sigma) was used to detect bystander activation. At the end of the coculture period, cells were stained with anti-CD4, anti-KLRG1, anti-CD107a, anti-CD25, anti-CD134, and anti-granzyme B antibodies as described above.

### Multicolor immunofluorescence

Orbital tissues were collected from GO and inflammatory pseudotumor (IP) patients at the First Affiliated Hospital of Xi’an Jiaotong University and Tangdu Hospital of Airforce Medical University. Tissue specimens from representative lesions were collected and fixed. Immunofluorescence was performed on 3-µm serial sections of paraffin-embedded tissue after dewaxing, antigen retrieval and blocking nonspecific binding. The primary antibodies used included an anti-CD4 antibody (Servicebio, GB13064-1, diluted at 1:50 or 1:800), anti-CD8 alpha rabbit polyclonal antibody (Servicebio, GB11068, diluted at 1:1,000) and anti-GZMB antibody (Abcam, UN, ab4059, diluted at 1:400). Secondary antibodies were used according to the species origin of the associated primary antibody. Multicolor immunohistochemistry data were collected on a fluorescence microscope (Nikon Eclipse C1) connected to an imaging system (Nikon DS-U3). Multispectral imaging was performed using CaseViewer 3.3 at 40× magnification. Image analysis was performed using ImageJ version 2.3. Between 5–10 high-power fields were evaluated per patient sample depending on tissue size to quantitate the average percentages of CD4+, CD8+ and CD4+ GZMB+ T cells.

Sorted CD4+ KLRG1+ T cells from GO patients were prepared by cytospin and then incubated with the primary antibodies mouse anti-CD4 (1:200, clone MT310, Santa) and rabbit anti-GZMB (1:200) in humidified boxes overnight at 4 °C after blocking with 0.01 M PBS containing 3% BSA for 30 min. Then, the cells were incubated with secondary antibodies conjugated to Alexa Fluor 594 (anti-rabbit IgG, Invitrogen) or Alexa Fluor 488 (anti-mouse IgG, Invitrogen) in the dark for 1 h at room temperature. After incubation with Hoechst 33258 (Sigma) to stain all nuclei, cells were examined and imaged under a confocal microscope (LSM 800, Zeiss, Oberkochen, Germany).

### Quantitative real-time PCR

Total RNA was extracted from approximately 100,000–500,000 sorted CD4+ KLRG1+ or naïve CD4+ T cells using the Direct-zol RNA Microprep Kit (Zymo Research). cDNA was synthesized using the Prime Script Master Mix Kit (TaKaRa) according to the manufacturer’s protocol, followed by quantitative real-time PCR analysis (SYBR green; TaKaRa). GAPDH mRNA expression was used as the normalization control. The primers used were ThPOK (F: 5’-GTCCCCAGAGCTACGAACC-3’, R: 5’-AGCTTAGGTAGGCCATCAGGT-3’), Runx3 (F: 5’-CAGAAGCTGGAGGACCAGAC-3’, R: 5’-GTCGGAGAATGGGTTCAGTT-3’), and GAPDH (F: 5’-ATGTTCGTCATGGGTGTGAA-3’, R: 5’-GTCTTCTGGGTGGCAGTGAT-3’).

### Statistical analysis

The results are shown as the mean ± standard error of the mean (s.e.m.) or median (quartile 1, quartile 3) depending on normality. Boxplots are shown with the plot center and box corresponding to the median and interquartile range (IQR), respectively, and include individual data points. Comparisons between two groups were performed using Student’s *t* tests (normal distribution) and the Mann–Whitney test (nonnormal distribution). Statistical significance was accepted at *P* < 0.05. All statistical analyses were performed and images were generated using R (version 3.4.0) and GraphPad Prism (version 7.0), respectively.

## Results

### CD4**+** T cells are the most abundant lymphocytes that infiltrate GO orbital tissues

In GO, the immune cells infiltrating the extraocular muscles, lacrimal glands, and adipose tissues are primarily CD4+ T cells,^43,44^ which produce cytokines and chemokines, including IFN-γ, TNF and C–X–C motif ligands.^45,46^ We applied immunofluorescence staining to evaluate orbital tissues from GO patients and confirmed that the cell counts of CD4+ T cells were significantly higher than those of CD8+ T cells (*P* = 4.5 × 10^−8^, Student’s *t* test, Supplementary Fig. 1).

### Droplet-based scRNA-Seq of CD4**+** T cells from GO and GH patients

To further characterize CD4+ T cells in GO, we performed droplet-based scRNA-Seq^47^ of CD4+ T cells from the peripheral blood of patients, including treatment-naïve GH patients (newly diagnosed GH patients without treatment) (*n* = 3), treatment-naïve GO patients (newly diagnosed GO patients without treatment) (*n* = 6), and treatment withdrawal GO patients (*n* = 4) (Supplementary Tables 1–2). After QC,^48^ 18,474 unique transcripts were obtained from 85,265 cells, in which a median number of 1172 genes could be detected (Supplementary Table 3).

**Fig. 1:**
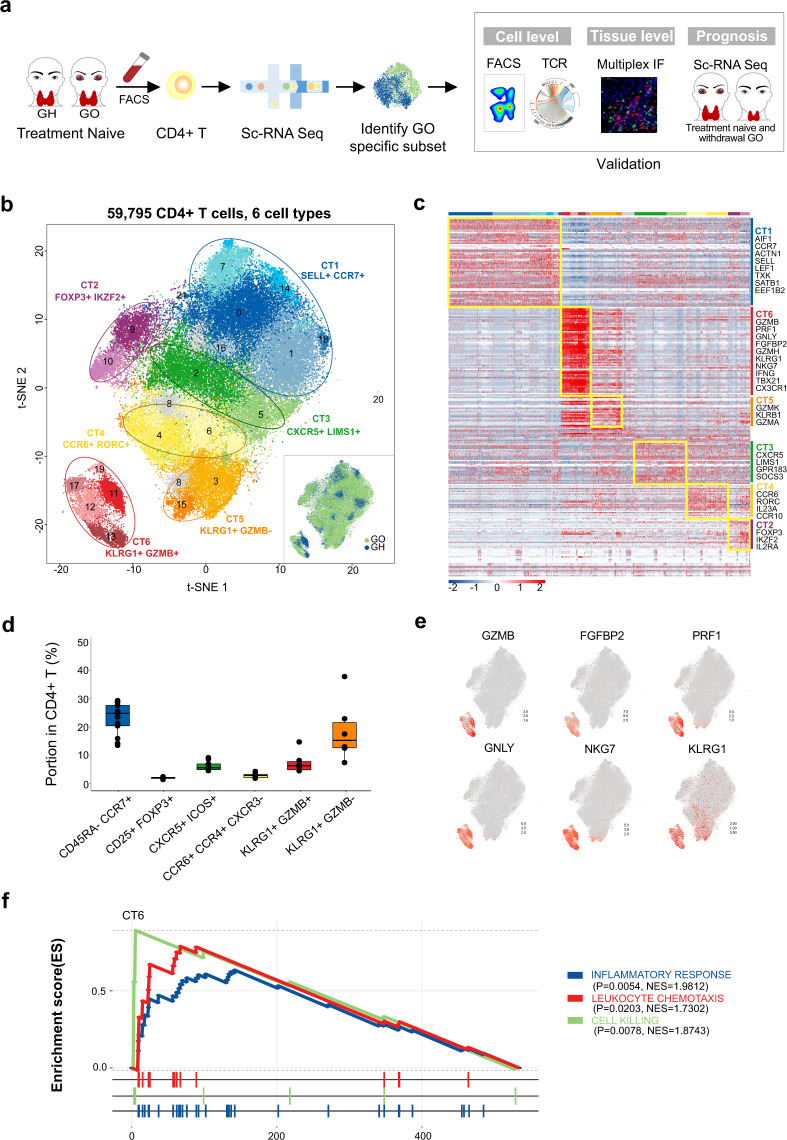
Identification of the GO-specific T cell type: a novel CD4+ CTL subtype with properties of chemotaxis and inflammation. **a** Flow chart of the overall study design. **b** Graph-based clustering and the t-SNE algorithm were applied to 59,795 CD4+ T cells from the peripheral blood of 6 GO and 3 GH patients. Clusters denoted by the same color scheme were labeled with inferred cell types. The clusters in the bottom right denoted by green and blue are labeled with disease types. **c** Heat map from single-cell analysis via expression data of the top ten genes differentially expressed for each cluster with cells grouped into cell types (indicated by colored bars at the top). Key genes for each cell type are shown on the right margin. **d** Expression level of canonical lineage markers for terminal effect cytotoxic T cells (CT6) across 59,795 CD4+ T cells illustrated in t-SNE plots. **e** GSEA plots for the indicated gene sets in the transcriptome of CT6 from GO versus GH. The positive enrichment score indicated a positive correlation with the GO group. NES, normalized enrichment score

To identify the specific CD4+ T cell types associated with GO progression, we merged data across treatment-naïve GO (*n* = 6) and GH (*n* = 3) patients and performed graph-based clustering (Fig. 1a). t-SNE analysis identified 22 clusters, most of which were shared across multiple patients (Fig. 1b, Supplementary Figs. 2–4). Based on the expression of signature genes in each cluster and canonical lineage markers,^49,50^ we identified six CD4+ T cell types (CTs), which largely represented central memory CD4+ T cells (CT1), regulatory T cells (CT2), follicular CD4+ T cells (CT3), Th17 cells (CT4), intermediate differentiated CTLs (CT5) and terminal effector CTLs (CT6)^16,51^ (Fig. 1b, c; Supplementary Fig. 5; Supplementary File 1). The remaining cells were labeled as unknown cells (4.48%). Flow cytometry analysis of CD4+ T cells from 16 independent patients confirmed both the above cell types and their corresponding frequencies^52^ (Fig. 1d; for details related to the cell markers and frequencies of the above cell types, see Supplementary Tables 4–6).

### GO-specific T cell type: a novel proinflammatory CD4+ CTL subtype with chemotactic and inflammatory properties

Although most cell types exhibited considerable overlap between GO and GH, CT6 exhibited disease-specific subtypes (Fig. 1b, Supplementary Fig. 7). While both CT5 and CT6 belonged to CD4+ CTLs, CT6 exhibited a unique gene profile containing KLRG1, GZMB, PRF1, GNLY, CX3CR1, FGFBP2, CCL4, CCL5 and IFNG, suggesting a terminal effector stage with novel chemotactic and inflammatory properties (Fig. 1e, Supplementary Fig. 5). Consequently, we performed GSEA of CT6, and Gene Ontology analysis revealed cell killing, leukocyte chemotaxis and inflammatory response as the top enriched signatures in GO patients (Fig. 1f, Supplementary Fig. 8, Supplementary File 2). Hallmark gene sets further supported the increased inflammatory response of CT6 in GO (Supplementary Fig. 9). Thus, we identified a novel CD4+ CTL subtype characterized by chemotaxis and inflammation as a GO-specific T cell type.

### Immunohistochemistry confirmed the presence of granzyme-releasing CD4**+** CTLs in GO orbital tissues

**Fig. 2:**
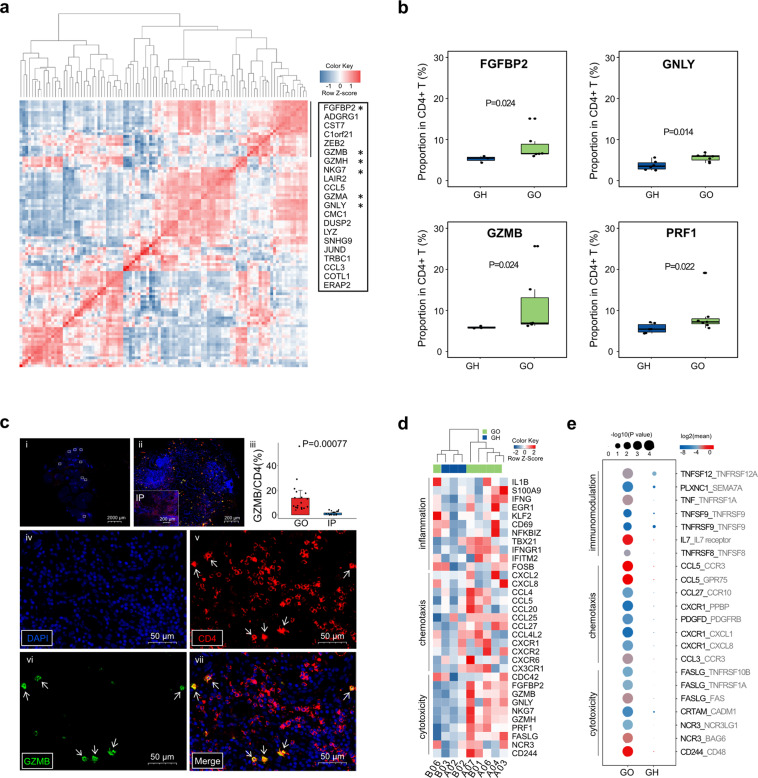
The characteristics of GO-specific CD4+ T cell types included cytotoxicity, chemotaxis and inflammation. **a** Pearson correlation with cytotoxic expression in CT6 cells. Pearson correlation plot showing the coexpression of the top 100 CT6-specific transcripts in GO. The black solid line shows a cluster of transcripts showing a high correlation; the list of these transcripts is shown in the text box on the right, and stars highlight the genes with cytotoxic functions. **b** Boxplots showing the proportions of the cytotoxic molecules FGFBP2, GNLY, GZMB and PRF1 in CD4+ T cells by flow cytometry (*n* = 7 in GO; and *n* = 6 in GH). Boxplots with the plot center and box corresponding to the median and IQR, respectively, and include individual data points. **c** Representative example of orbital tissue from GO patients and inflammatory pseudotumor (IP) patients by fluorescent multiplex immunohistochemistry showing coexpression of CD4 and GZMB. (i) Low-magnification image of whole orbital tissue. (ii) Image at 10× magnification with a proportion of GZMB in CD4+ T cells higher in GO than in IP patients. (iii) Quantification of the GZMB proportion in CD4+ cells for *n* = 6 independent cases, including representative cases. Error bars are mean ± SEM. Data points, each representing one case, are shown by solid circles. (iv-vii) Images at 40× magnification of a single section stained for DAPI, CD4 and GZMB, illustrating coexpression of CD4 and GZMB (small arrows). Information on the other samples is shown in Supplementary Fig. 11. **d** Hierarchical clustering of CT6 among 6 GO and 3 GH patients by genes involved in inflammation, chemotaxis and cytotoxicity activities. The heatmap shows, for each gene, the scaled average expression over all cells of each patient. **e** Selected ligand–receptor interactions between CT6 and CT4 cells in GO and GH, respectively. The black and gray fonts represent the ligands or receptors from CT6 and CT4, respectively. *P* values are indicated by the circle size. The means of the average expression levels of interacting molecules in CT6 and CT4 are indicated by color

A more detailed coexpression analysis of specific transcripts in the proinflammatory CD4+ CTL subtype in GO revealed that cytotoxicity signature genes were highly correlated (Fig. 2a). Further flow cytometry analysis of PBMCs from 13 independent patients (6 GH patients and 7 GO patients) confirmed that the protein levels of cytotoxic molecules (FGFBP2, GNLY, GZMB and PRF1) in CD4+ T cells were significantly increased in the cells from GO patients (*P* = 0.024, 0.014, 0.024 and 0.022, respectively, Student’s *t* test; Fig. 2b, Supplementary Fig. 10). Given that GZMB expression was significantly higher in PBMCs from GO patients than in those from GH patients, we further performed immunofluorescence analysis of orbital tissues from GO and IP patients. We confirmed that the expression level of GZMB in CD4+ T cells was higher in GO patients than in IP patients (*P* = 0.00077, Fig. 2c, Supplementary Fig. 11), suggesting that these cells are highly specific to GO and may have a role in the pathogenesis of GO.

### The novel proinflammatory CD4+ CTL subtype is the main source of chemokines and inflammatory cytokines in GO

In addition to the cytotoxicity described above, the GO-specific CD4+ CTL subtype expressed relatively high levels of chemotactic and inflammatory molecules, including CCL4, CCL5, CXCL8, CX3CR1, IL1B and IFNG (Fig. 2d). Furthermore, flow cytometry revealed that the expression levels of CX3CR1 and IFNG in GO-specific CD4+ CTLs were increased compared with those in other nonspecific CD4+ CTLs (CT5) (*P* = 0.061 and 0.016; Supplementary Fig. 12). Interaction analysis^52^ showed that proinflammatory CD4+ CTLs and Th17 cells (CT4) in GO expressed increased levels of chemotactic ligands (CCL3, CCL5 and CCL27) and receptors (CCR3 and CCR10), respectively (Fig. 2e). Immunomodulatory and cytotoxic pairs were also enriched in GO (Fig. 2e). Since Th1-like cytokines have been suggested to play vital roles in perpetuating orbital inflammation and remodeling,^2,21^ we investigated the canonical gene expression of Th1 cells and revealed the unique expression of TBX21, EOMES, IFNG and GZMB in GO-specific CD4+ CTLs (Supplementary Fig. 13). Together, our data suggested that the GO-specific CD4+ CTL subtype was the main source of the chemokines and proinflammatory cytokines that initiate and perpetuate the autoimmune-inflammation cascade in GO.

### GO relapse was related to increased levels of chemotactic and cytotoxic molecules in the novel proinflammatory CD4+ CTL subtype

**Fig. 3:**
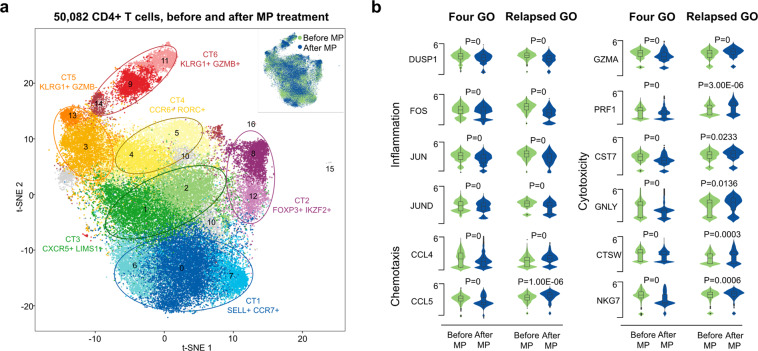
GO relapse was related to increased levels of chemotaxis and cytotoxicity molecules in proinflammatory CD4+ CTLs after treatment. **a** Graph-based clustering and the t-SNE algorithm were applied to 50,082 CD4+ T cells from 4 GO patients before and after treatment (*n* = 8). Clusters denoted by the same color scheme were labeled with inferred cell types. The clusters in the top right denoted by green and blue were labeled before and after treatment, respectively. **b** Violin plots showing the single-cell expression pattern of the indicated marker genes involved in inflammation, chemotaxis and cytotoxicity of CT6 from four GO patients and one relapsed GO patient (A04) before and after treatment. The shapes represent the distribution of cells based on the gene expression

Due to the potential roles of cytotoxicity, chemotaxis and inflammation in GO progression, we examined the treatment effects of methylprednisolone (MP) on these three pathways by scRNA-Seq of CD4+ T cells from four GO patients before and after treatment (Fig. 3a, Supplementary Figs. 14–15, Supplementary File 3). We observed that the levels of cytotoxicity-, chemotaxis- and inflammation-related genes were significantly downregulated after MP treatment in the proinflammatory CD4+ CTL subtype (Fig. 3b). Of note, one (A04) of the four GO patients did not respond well to MP and relapsed four months after treatment withdrawal. We analyzed the proinflammatory CD4+ CTL subtype in this patient and found significantly increased levels of chemotaxis- and cytotoxicity-related molecules after MP withdrawal, although inflammation-related molecules were significantly ameliorated (Fig. 3b).

### Functional CD4**+** CTLs were contained in the CD4**+** KLRG1**+** T cell population

**Fig. 4:**
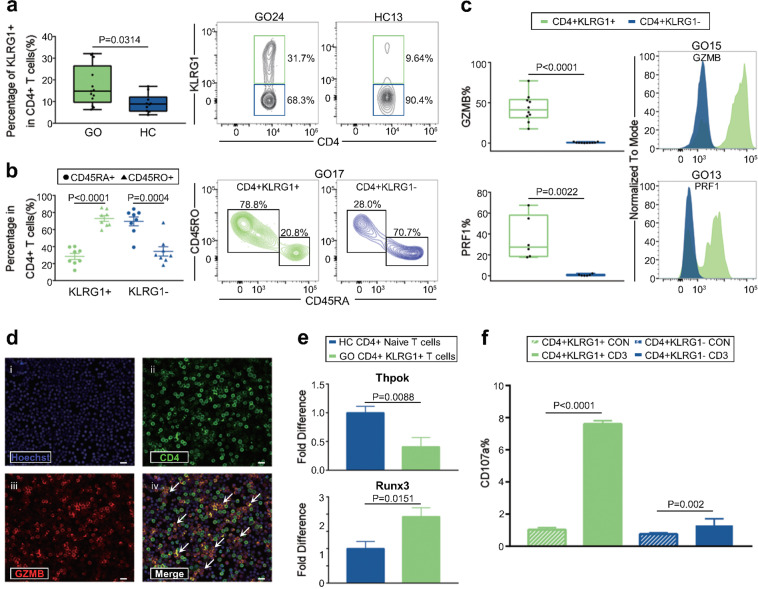
Functional CD4+ CTLs were contained in CD4+ KLRG1+ T cells. **a** Boxplot (left) showing the proportion of KLRG1+ CD4+ T cells (*n* = 13 in GO and *n* = 10 in HC). Boxplot with the plot center and box corresponding to median and extremum, respectively and include individual data points. Representative flow cytometry plots (middle and right) of CD4+ KLRG1+ T cells gated in CD4+ T cells from samples GO24 and HC13. The numbers denote the percentage of cells in each rectangular gate. **b** The percentage of CD45RA+ (round) and CD45RO+ (triangle) cells in CD4+ KLRG1+ (green) and CD4+ KLRG1− (blue) T cells, respectively (*n* = 8 in GO). Data are means ± SEMs and individual values. Representative flow cytometry plots (middle and right) of CD45RA+ and CD45RO+ cells in CD4+ KLRG1+ and CD4+ KLRG1− T cells in sample GO17. The numbers denote the percentage of cells in each rectangular gate. **c** Boxplots (left) showing the ratio of GZMB (upper) and PRF1 (bottom) in CD4+ KLRG1+ (green) and CD4+ KLRG1− (blue) T cells (*n* = 10 in GO). Boxplots with plot center and box corresponding to median and extremum, respectively and include individual data points. Representative histograms (right) of GZMB (upper) and PRF1 (bottom) in CD4+ KLRG1+ (green) and CD4+ KLRG1− (blue) T cells in samples GO15 and GO13, respectively. **d** Representative example of CD4 and GZMB coexpression (small arrows) detected by confocal microscopy with staining for (i) Hoechst, (ii) CD4, (iii) GZMB, and (iv) merged CD4+ KLRG1+ T cells from GO patients. Scale bars 20 μm. **e** Bar plots showing the levels of Thpok (upper) and Runx3 (bottom) in CD4+ KLRG1+ T cells (green) from GO patients and CD4+ naïve T cells (blue) from HCs (*n* = 13 in GO; and *n* = 10 in HC). Error bars show SEM. **f** Bar plot showing the percentage of CD107a in CD4+ KLRG1+ T cells (green) and CD4+ KLRG1− T cells (blue) from GO patients after treatment with blank (control, slash) and anti-human CD3 (filled) antibodies for 6 h. Error bars show SEM. The data are representative of at least three biological replicates

The surface protein marker KLRG1, which is localized in CD8+ T cells and NK cells,^53,54^ was highly expressed by CD4+ CTLs (Fig. 1d). To test (i) whether KLRG1 can be used as a potential marker for CD4+ CTLs and (ii) identify the GO-specific CD4+ CTLs with unique cytotoxic (GZMB and PRF1), chemotactic (CX3CR1, CCL4, CCL5) and inflammatory (IFNG) signatures enriched in this cell compartment, we isolated CD4+ KLRG1+ T cells from GO patients. To test whether CD4+ KLRG1+ cells are related to GO, the proportion of CD4+ KLRG1+ T cells in GO patients was compared with that in healthy controls (HCs), and the proportion was significantly increased in GO patients (8.88% (IQR: 5.625%, 12.08%) vs 14.8% (IQR: 9.68%, 26.45%), respectively, *P* = 0.0314, Mann–Whitney test, Fig. 4a). Flow cytometry analysis of CD4+ KLRG1+ T cells also confirmed the frequencies of CD4+ CTLs measured by scRNA-Seq (Supplementary Tables 4–5).

CD4+ CTLs have been identified in differentiated T cell subsets, such as effector memory T cells.^46,55^ Likewise, our results indicated that CD4+ KLRG1+ T cells from GO patients exhibited a CD45RO+ memory phenotype rather than a CD45RA+ naïve phenotype (72.99%±3.393% vs. 28.4%±3.566%, *P* < 0.0001, Student’s *t* test; Fig. 4b). Compared with CD4+ KLRG1− T cells, CD4+ KLRG1+ T cells showed significantly increased expression of the cytotoxic molecules GZMB and PRF1 (CT6 unique) in GO patients (0.365% (IQR: 0.2975%, 0.885%) vs 41.2% (IQR: 31.38%, 53.95%) for GZMB, *P* < 0.0001; 0.465% (IQR: 0.062%, 1.298%) vs 27.4% (IQR: 18.45%, 58.05%) for PRF1, *P* = 0.0022, Mann–Whitney test, Fig. 4c). Next, we applied immunofluorescence analysis to confirm that GZMB was present in a large fraction of CD4+ KLRG1+ T cells (Fig. 4d). In addition, the levels of chemotactic molecules, including CX3CR1, CCL4 and CCL5, together with that of the inflammatory molecule IFNG in CD4+ KLRG1+ T cells were elevated compared with the levels in CD4+ KLRG1− T cells in GO patients according to flow cytometry (Supplementary Fig. 16).

It was reported that increased expression of the transcription factor Runx3 and loss of Thpok are required for the reprogramming and maturation of functional CD4+ CTLs in mice.^56^ Likewise, our results showed that CD4+ KLRG1+ T cells from GO patients presented lower expression of Thpok (*P* = 0.0088, Student’s *t* test) but higher expression of Runx3 (*P* = 0.0151, Student’s *t* test) than naïve CD4+ T cells from an HC^30^ (isolated with a Miltenyi human naïve CD4+ T cell isolation kit, and >90% of the cells obtained were CD45RA+ CCR7+) (Fig. 4e).

Furthermore, to verify whether CD4+ KLRG1+ T cells possess cytotoxic molecule release functions, a degranulation assay was performed. Lysosome-associated membrane glycoprotein (CD107a), which is localized in lysosomes, is expressed on the cell surface after granule secretion in CD8+ CTLs.^57,58^ In our study, upon in vitro stimulation with an anti-human CD3 antibody, CD4+ KLRG1+ T cells underwent degranulation with a significant increase in the surface expression of CD107a (*P* < 0.0001, Student’s *t* test, Fig. 4f). Overall, CD4+ KLRG1+ T cells from GO patients were functional CD4+ CTLs with GZMB and PRF1 expression (CT6 unique).

### CD4**+** KLRG1**+** CTLs in GO exhibited marked clonal expansion

**Fig. 5:**
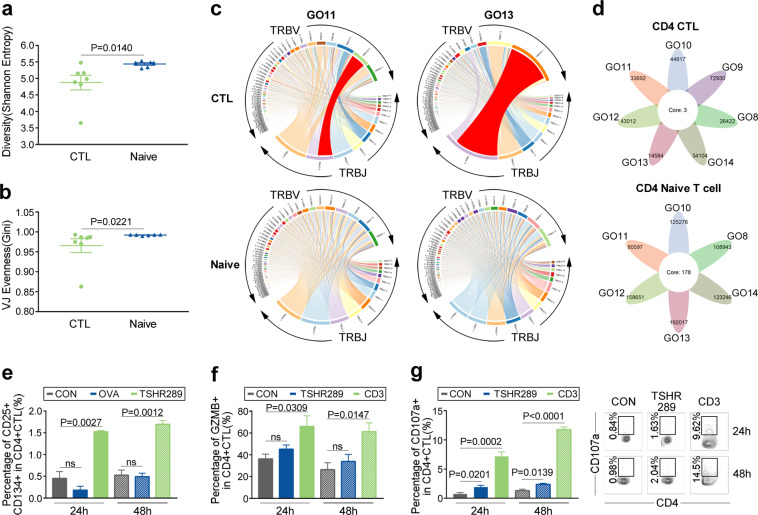
CD4+ KLRG1+ CTLs in GO exhibited marked clonal expansions. Whisker plot showing TCR VJ diversity (**a**) and evenness (**b**) by the Shannon entropy and Gini coefficient in CD4+ KLRG1+ CTLs (green, *n* = 7) and CD4+ naïve T cells (blue, *n* = 6) from GO patients. Data are presented as the mean with SEM of individual values. **c** TRBV and TRBJ gene segment usage and V-J recombination are illustrated by circos plots in samples GO11 and GO13. The TRBV and TRBJ genes were arranged clockwise in the order of their frequency from low to high. A VJ recombination is illustrated by colored curved paths whose thickness represents their frequencies in the TCR repertoires. **d** Flower plots present the amount of overlap (core) and individual overlap (petal) in TCR clonotypes in CD4+ KLRG1+ CTLs (upper, *n* = 7) and CD4+ naïve T cells (bottom, *n* = 6), respectively. **e–g** Isolated CD4+ T cells and mitomycin-treated non-CD4+ T cells were cocultured at a 1:3 ratio with blank control (gray), 25 μg/mL human TSHR289 protein (green or blue), 25 μg/mL ovalbumin (OVA) (blue) or 10 μg/mL anti-human CD3 (green) for 24 h (filled) and 48h (slash). Comparisons of the CD25+ CD134+ (**e**), GZMB+ (**f**) and CD107a+ (**g**) ratios in CD4+ KLRG1+ CTLs are shown by bar plots to detect the TSHR-specific response. Error bars show the SEM. All data are representative of at least three biological replicates. Representative flow cytometry plots (right) of the CD107a+ cell ratio in CD4+ KLRG1+ CTLs after coculturing for 24 h and 48 h in the control, TSHR289 protein and anti-human CD3 groups. The numbers denote the percentage of cells in each rectangle

Given that memory CD4+ CTLs are largely generated after antigen exposure, such as dengue virus exposure,^59^ we speculated that clonal expansions exist in the CD4+ KLRG1+ T cell population. Thus, TCR sequencing (TCR-Seq) of circulating CD4+ KLRG1+ CTLs from GO patients was performed. As expected, CD4+ KLRG1+ T cells from GO patients had significantly decreased diversity and evenness compared with naïve CD4+ T cells (*P* = 0.014, *P* = 0.0221, Mann–Whitney test, Fig. 5a, b). Specifically, in GO samples, the top V-J recombination was often observed in a large percentage of CD4+ KLRG1+ CTLs, such as in GO11 (11.13%) and GO13 (34.06%) (Fig. 5c). The expansion of certain CD4+ KLRG1+ T cell clones might be a specific immune response to autoantigen exposure. It was reported that CD4+ CTLs are heterogeneous across patients.^16^ Thus, we analyzed the overlap in TCR clonotypes in CD4+ KLRG1+ CTLs and naïve CD4+ T cells across 7 GO samples, and only 3 clonotypes were shared among the CD4+ CTLs, while 178 clonotypes were shared among the naïve CD4+ T cells (Fig. 5d).

TSHR serves as the primary autoantigen in GO based on evidence of the close temporal correlation of GO and GH, a GO animal model and the relationship of disease activity with anti-TSHR antibodies.^11,60–62^ To explore whether TSHR-specific T cells exist in the CD4+ KLRG1+ CTL population, an MLR with TSHR 289 was performed. Compared with control or irrelevant antigen ovalbumin (OVA) stimulation, after stimulation with TSHR289 for 24 h or 48 h, CD4+ KLRG1+ CTLs were significantly activated, as indicated by the increased level of CD25+ CD134+ cells^63,64^ (0.45% ± 0.16% vs 1.527% ± 0.02667%, *P* = 0.0027 at 24 h, 0.5267% ± 0.1167% vs 1.697% ± 0.08333%, *P* = 0.0012 at 48 h, Fig. 5e). Increased expression of GZMB was also detected in CD4+ KLRG1+ CTLs upon TSHR289 stimulation, although there was no significant difference compared to control stimulation due to the potential release of granules (Fig. 5f). As expected, the expression of CD107a in the TSHR289 group was significantly enhanced compared with that in the blank control group (1.91% ± 0.2949% vs. 0.735% ± 0.2303% at 24 h, *P* = 0.0201; 2.368% ± 0.194% vs. 1.295 ± 0.2451% at 48 h, *P* = 0.0139, Student’s *t* test, Fig. 5g). In summary, these results revealed that CD4+ KLRG1+ CTLs, which exhibited clonal expansion, contained TSHR-specific T cells and that CD4+ KLRG1+ CTLs have great potential to drive the progression of GO.

## Discussion

CD4+ CTLs likely play important roles in autoimmune-mediated inflammatory disorders; however, little is known about their functional properties and heterogeneity. Our results showed that cytotoxicity- and chemotaxis-related genes (GZMA, GZMM, CST7, KLRG1, CTSW, CCL4 and CCL5) were distributed across two CD4+ T cell subsets, CT5 and CT6. After comparing the two clusters, CT6 was distinguished by the expression of a strong unique signature that included GZMB, GZMH, PRF1, GNLY, CX3CR1 and FGFBP2, whereas CT5 was marked by only GZMK and CXCR3. Coordinated high expression of PRF1, GNLY, GZMB and the chemokine receptor CX3CR1 was associated with markers of late effector memory T cell differentiation. However, low PRF1 and high GZMK and GZMA levels are linked to the intermediate differentiation stage.^65^ Thus, CT6 was in line with the greater cytotoxic potential observed in a terminal effector state compared to the intermediate differentiated state of CT5.

In our study, the novel CD4+ CTL subtype with chemotactic and inflammatory characteristics (CT6) was specific for GO. These cells were characterized by cytotoxicity (*GZMB, KLRG1, GZMH, PRF1, GNLY and FGFBP2*), chemotaxis (*CCL4, CCL5 and CX3CR1*) and inflammation (*IL-1B and IFNG*). First, the fractalkine receptor CX3CR1 is the core signature for not only memory CD8+ CTLs^66^ but also CD4+ helper T cells that provide protective immunity against infection or persistent airway inflammation.^59,67^ CX3CR1 mediates leukocyte migration and adhesion, and recruitment of CTLs to sites of inflammation is achieved through CX3CL1-expressing cells.^68,69^ It has also been reported that CD34+ fibroblasts and immune cells traffic to orbital tissue by signaling through CCR5, which is the main receptor of CCL4 and CCL5.^70^ Second, orbital fibroblasts treated with IL-1β expressed elevated levels of IL-16 and IL-6, which are involved in chemoattractant activity specific for CD4+ T cells and regulation of adipocyte metabolism.^71^ Third, granzymes stored in CTLs directly influence inflammation by releasing proinflammatory cytokines, including IL-6, TNFα and IL-1β, which are focused on GZMA, GZMB, GZMK and GZMM.^32–34^ Granzymes can also degrade several extracellular matrix components, which contribute to inflammation and mediate tissue remodeling.^35,36^ Combined with the GO-specific and GO relapse-related features, this novel proinflammatory CD4+ CTL subtype has great potential for inducing the pathogenesis that drives the progression of GO.

The lack of a precise definition or sorting biomarker for CD4+ CTLs has greatly impaired the elucidation of their biology. To further explore the functions of CD4+ CTLs, we isolated CD4+ KLRG1+ T cells, as KLRG1 was highly expressed in CD4+ CTLs (CT5 and CT6). KLRG1 harbors an immune receptor tyrosine-based inhibitory motif in its cytoplasmic domain and exerts cytotoxic function,^72^ which makes it comparable to reported markers used to define CD4+ CTLs, including NKG2A and NKG2D.^73^ The correlation between increased KLRG1 expression and cytotoxic lymphocyte molecules such as GZMB and PRF1 has also been demonstrated.^74^ CD4+ KLRG1+ T cells were also confirmed to produce cytokines and have cytotoxic potential.^74^ Our findings revealed marked clonal expansion of CD4+ KLRG1+ CTLs from GO patients, suggesting a role for antigen-specific clonal expansion. These results were consistent with findings for CD4+ CTLs in other autoimmune diseases, such as systematic lupus erythematosus and type 1 diabetes mellitus.^7,30^ Notably, only 3 TCR clonotypes were shared in CD4+ KLRG1+ CTLs across 7 GO patients, and such limited common clonotypes implied the high-degree diversity of CD4+ KLRG1+ CTLs in accordance with the heterogeneity of CD4+ CTLs in supercentenarians.^75^ MLR analysis showed that after stimulation with the GO autoantigen TSHR289, the proportions of CD25+ CD134+ and CD107a+ cells were significantly increased. The activation and degranulation of CD4+ KLRG1+ CTLs might mediate cytotoxic molecule, proinflammatory cytokine and chemokine release and exacerbate orbital inflammation and remodeling. The mechanism underlying the inolvement of CD4+ KLRG1+ CTLs in the initiation and perpetuation of orbital inflammation still needs further exploration.

Due to the involvement of the CD4+ CTL subtype in the progression of GO, new treatment options could be developed. Although little has been reported on the development and shaping of the CD4+ CTL population, the mammalian target of rapamycin (mTOR) pathway is crucial in the differentiation of both CD4+ T cells and CD8+ CTLs, especially in regulating the decisions distinguishing effector and regulatory T cell lineage commitment.^76–80^ Moreover, it was recently found that an inhibitor of mTOR could selectively repress the expression of key CTL effector molecules, including GZMB, PRF and IFN-γ.^81^

Overall, our results revealed that the novel proinflammatory CD4+ CTL subtype with chemotactic and inflammatory characteristics was specific for GO. The demonstration of clonal expansion of these T cells by TCR-Seq, their localization at disease sites, and their relationship with disease relapse provide strong evidence for a pathogenic role for GZMB and IFN-γ-secreting CD4+ CTLs in GO. This proinflammatory CD4+ CTL subtype could migrate from the blood to orbital tissue and mediate orbital inflammation and remodeling according to its cytotoxic functions. To improve the treatment effect on GO, combining glucocorticoid therapy with targeting the causal mechanisms,^5,82^ especially cytotoxic pathways, is worth exploring in the future.

## Supplementary information

Supplementary Figures

Supplementary Tables

Supplementary File 1

Supplementary File 2

Supplementary File 3

## Data Availability

All scRNA-Seq raw sequencing data are available through the NCBI SRA accession PRJNA578302. Gene expression data for all clusters are available in Supplementary File 1. Cluster-specific gene expression data are available in Supplementary Files 2 and 3. The remaining data are available from the authors upon request.
