# Supplementary material for: A novel CD4+ CTL subtype characterized by chemotaxis and inflammation is involved in the pathogenesis of Graves’ orbitopathy
Source: Cell Mol Immunol. 2021 Jan 29;18(3):735–45. doi: 10.1038/s41423-020-00615-2 (PMC8027210; doi:10.1038/s41423-020-00615-2)
Supplement: Supplementary file 1 — Supplementary Figures [file 41423_2020_615_MOESM1_ESM.pdf]

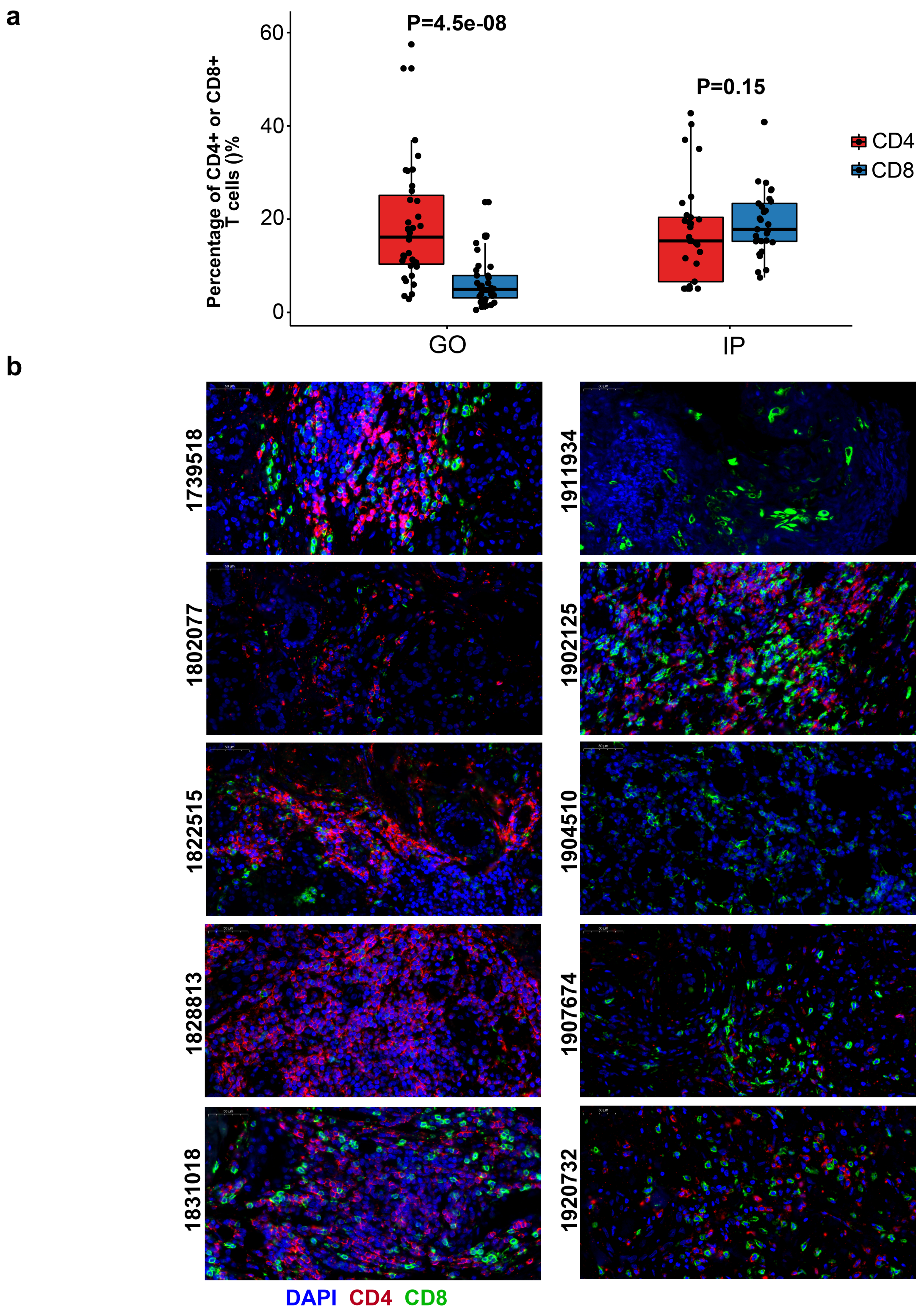

Supplementary Figure 1. Immunofluorescence of CD4 and CD8 in orbital tissues from GO and inflammatory pseudotumor (IP) patients. (a) The proportion of CD4 and CD8 in orbital tissues from five GO and five IP patients. Boxplots showed the median (center bar), the third and first quartiles (upper and lower edge of box, respectively). Data points, each representing one high-power field per sample, were shown by solid circles. (b) Representative high-power fields from five GO (left) and five IP (right) patients by fluorescent multiplex immunohistochemistry showing coexpression of CD4 (red) and CD8 (green).

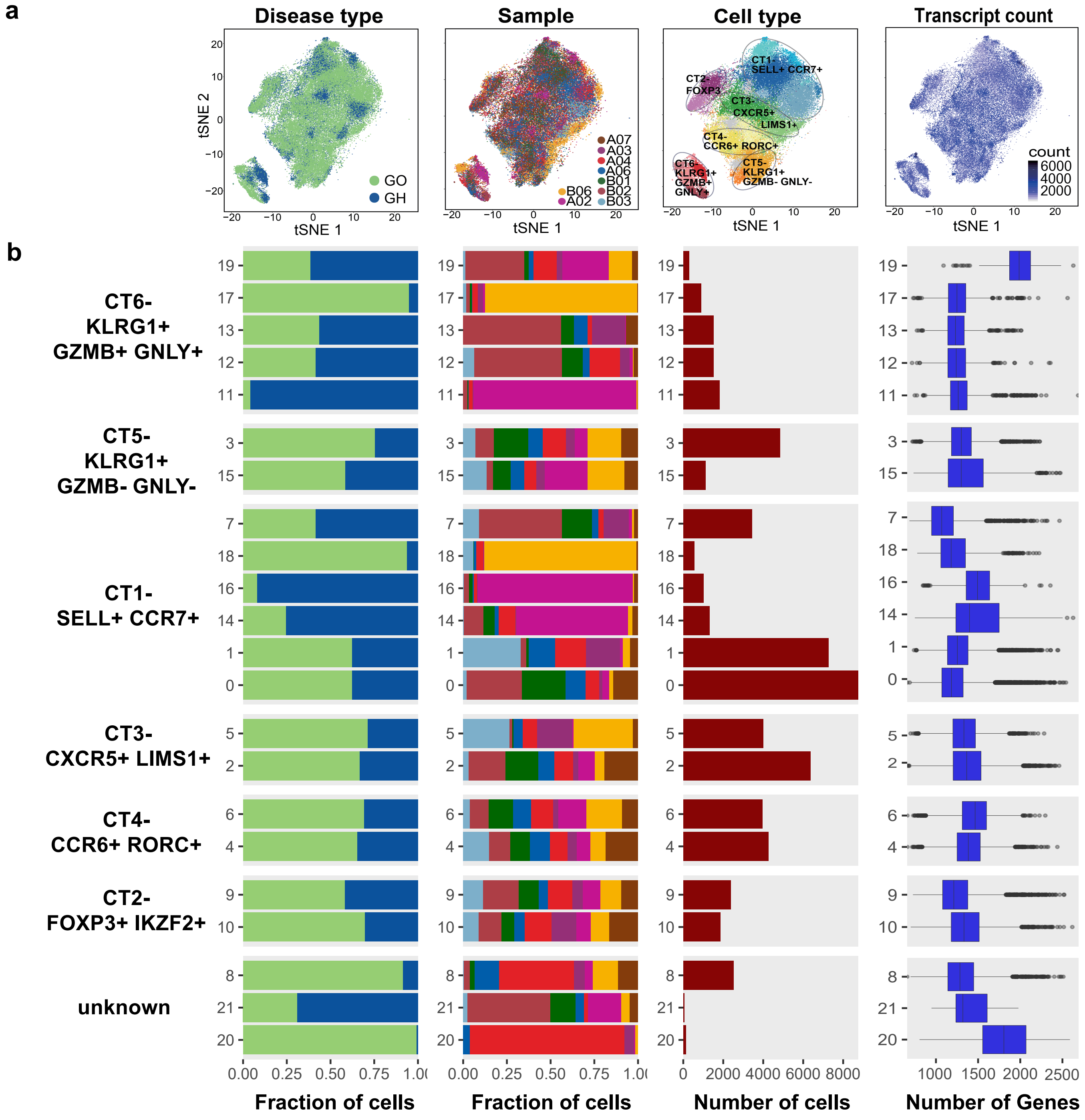

Supplementary Figure 2. Overview of the 59,795 single CD4+ T cells from treatment naïve GO and GH samples. (a) tSNE of the 59,795 cells profiling, with each cell colorcoded for (left to right): disease type (GO or GH), the corresponding patient, the cell types and the number of transcripts detected in that cell. (b) From left to right were the fraction of cells originating from 6 GO and 3 GH samples, the fraction of cells originating from each of the 9 patients, the number of cells and box plots of the number of transcripts (with plot center and box corresponding to median and IQR, respectively).

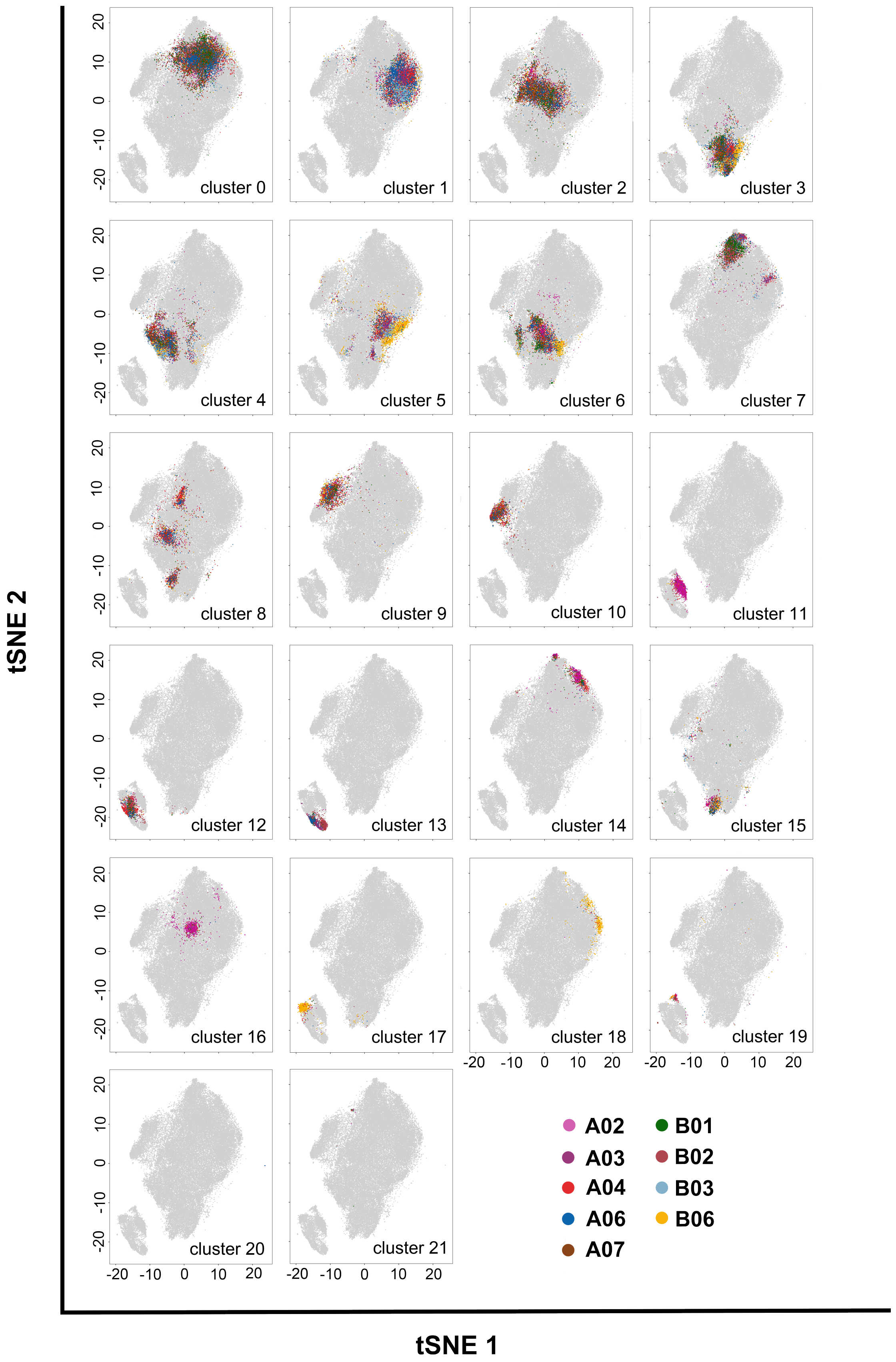

Supplementary Figure 3. The patient origins of each T cell were plotted by t-SNE for each cell cluster. Each dot corresponded to one single cell. And cells belonging to each cluster were colored according to patient origin on each subgraph.

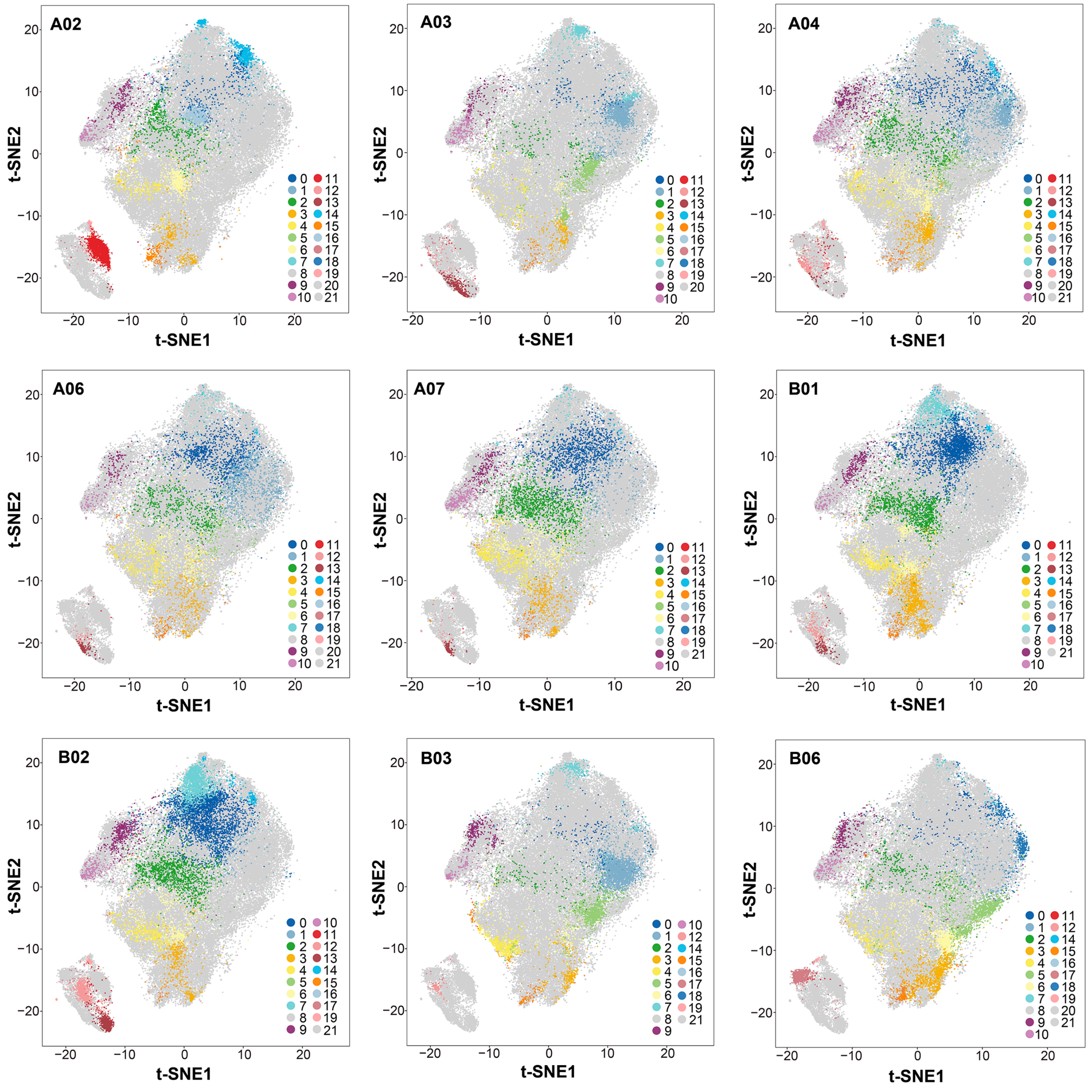

Supplementary Figure 4. The cluster origins of each T cell were plotted by t-SNE for each patients. Each dot corresponded to one single cell. And cells belonging to each patient were colored according to cluster origin on each sub-graph.

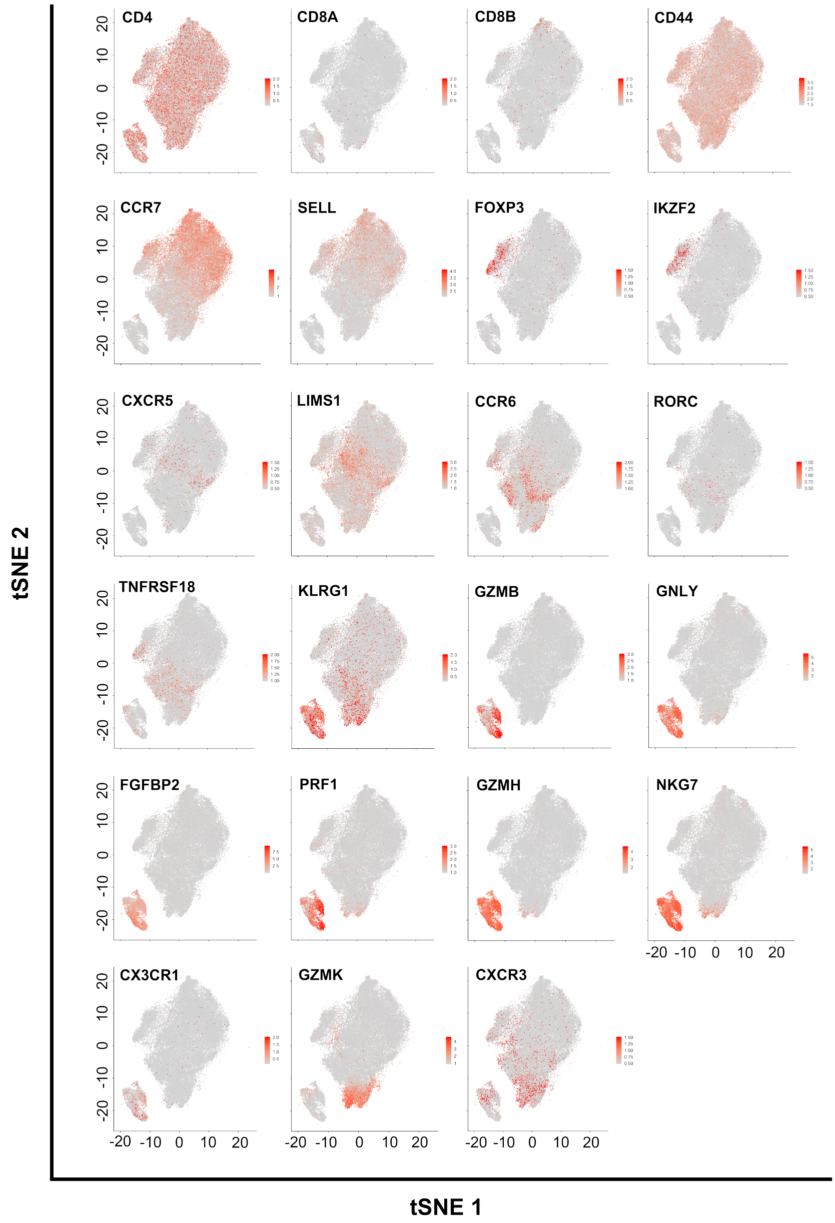

Supplementary Figure 5. Expression of canonical lineage markers for each cell types. CT1 mapped more closely to CD4+ central memory than naïve T cells, for they not only expressed high level of SELL, CCR7 and LEF1, but also CD69 and CD44. CT2 was close to regulatory T cells which was best defined by the functional markers FOXP3, IKZF2 and IL2RA. CT3 was consisted of cells with features of follicular CD4+ T cells, including the expression of CXCR5, ICOS, TIGIT, MAF, GPR183 and CD84. CT4 contained Th17 cells which would be identified by the unique expression of CCR6, RORC, IL23A and TNFRSF18. Cytotoxicity and chemotaxis related genes (GZMA, GZMM, CST7, KLRG1, CTSW, CCL4 and CCL5) were distributed across CT5 and CT6. Comparing the two clusters, CT6 were distinguished by the expression of a strong unique signature that includes GZMB, GZMH, PRF1, GNLY, CX3CR1 and FGFBP2, whereas CT5 were marked only by unique genes of GZMK and CXCR3. Coordinated high expression of PRF1, GNLY, GZMB and chemokine receptor CX3CR1 was associated with markers of late effector memory differentiation. Whereas, low PRF1 and high GZMK, GZMA are linked to intermediate differentiation stage. Thus, CT6 was in line with greater cytotoxic potential observed in a terminal effector state compared to intermediate differentiated CT5.

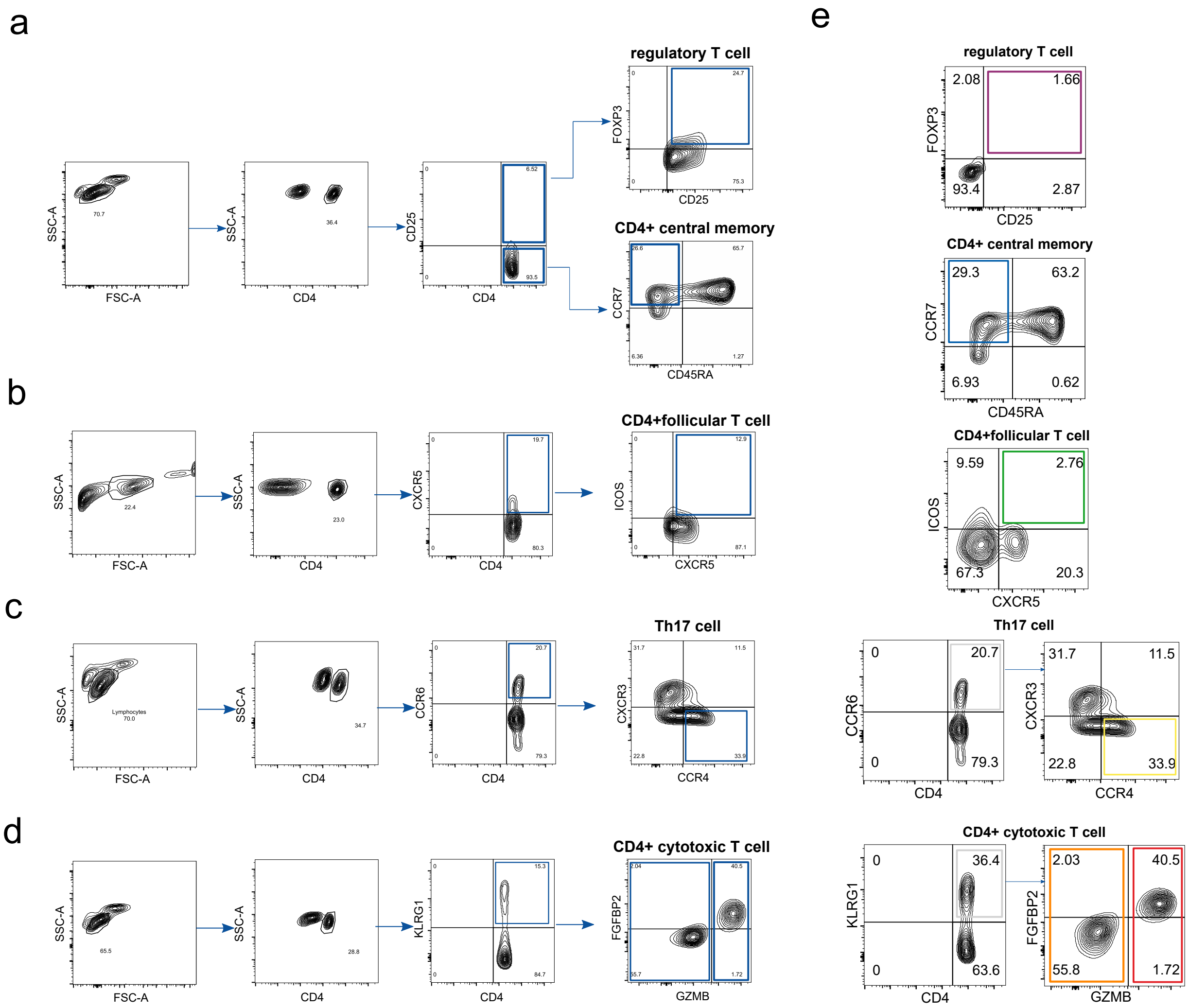

Supplementary Figure 6. Flow cytometry analysis of six CD4+ cell types. (a-d) Gating of CD4+ central memory T cells, Regulatory T cells, CD4+ Follicular T cells, Th17 cells, GZMB+ cytotoxic T and GZMB- cytotoxic T cells. (e) Flow cytometry analysis showed the surface expression of CCR7, CD45RA, CD25, CCR6, CCR4, CXCR3, CXCR5, ICOS, KLRG1 and FGFBP2, and intracellular expression of FOXP3 and GZMB in singlet-gated CD4+ cells in representative patients. The numbers denote the percentage of cells in each quadrant.

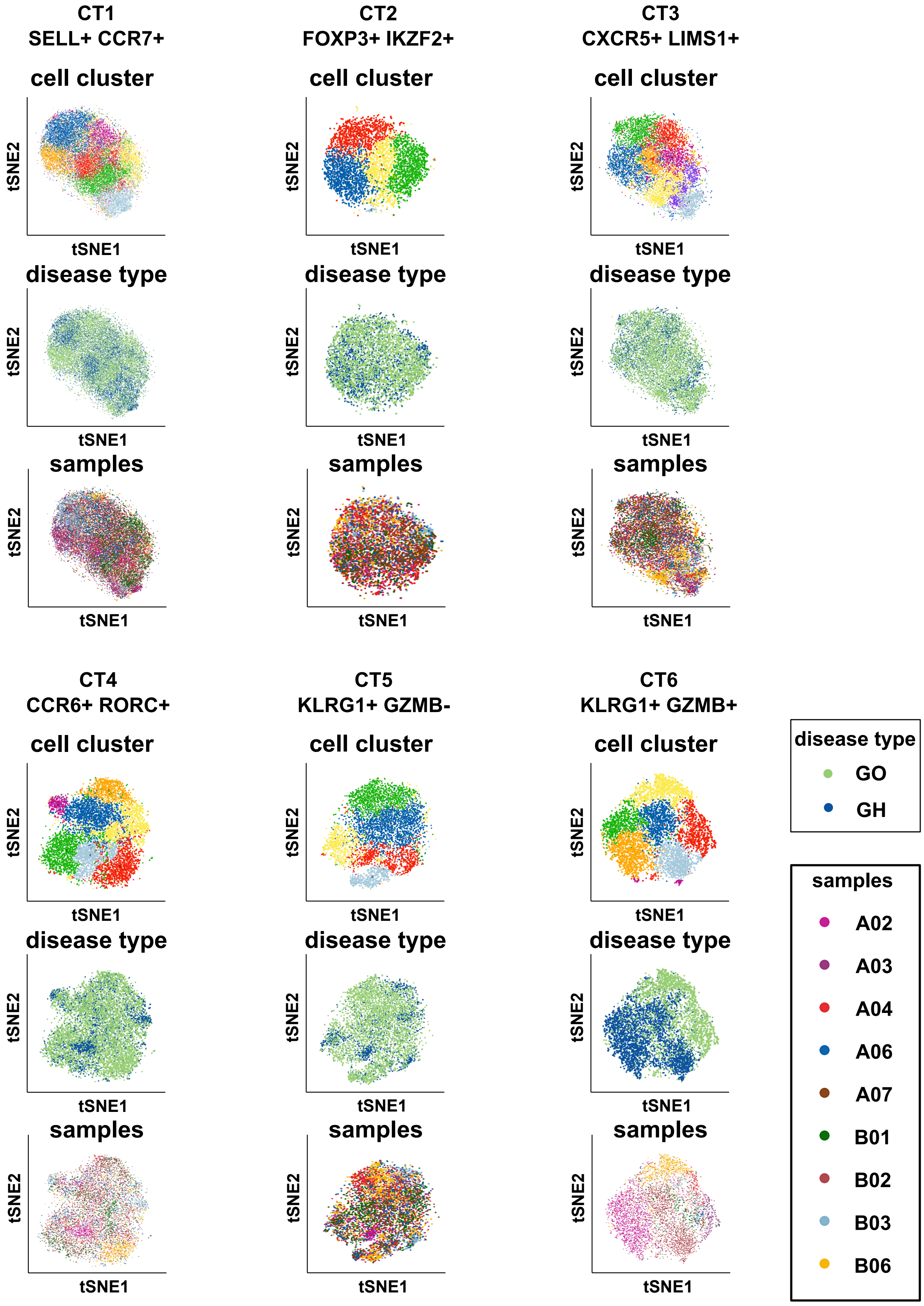

Supplementary Figure 7. Reclustering the cells from each cell type. Each cell colorcoded for (top to bottom): clusters, disease type (GO or GH) and the corresponding patient.

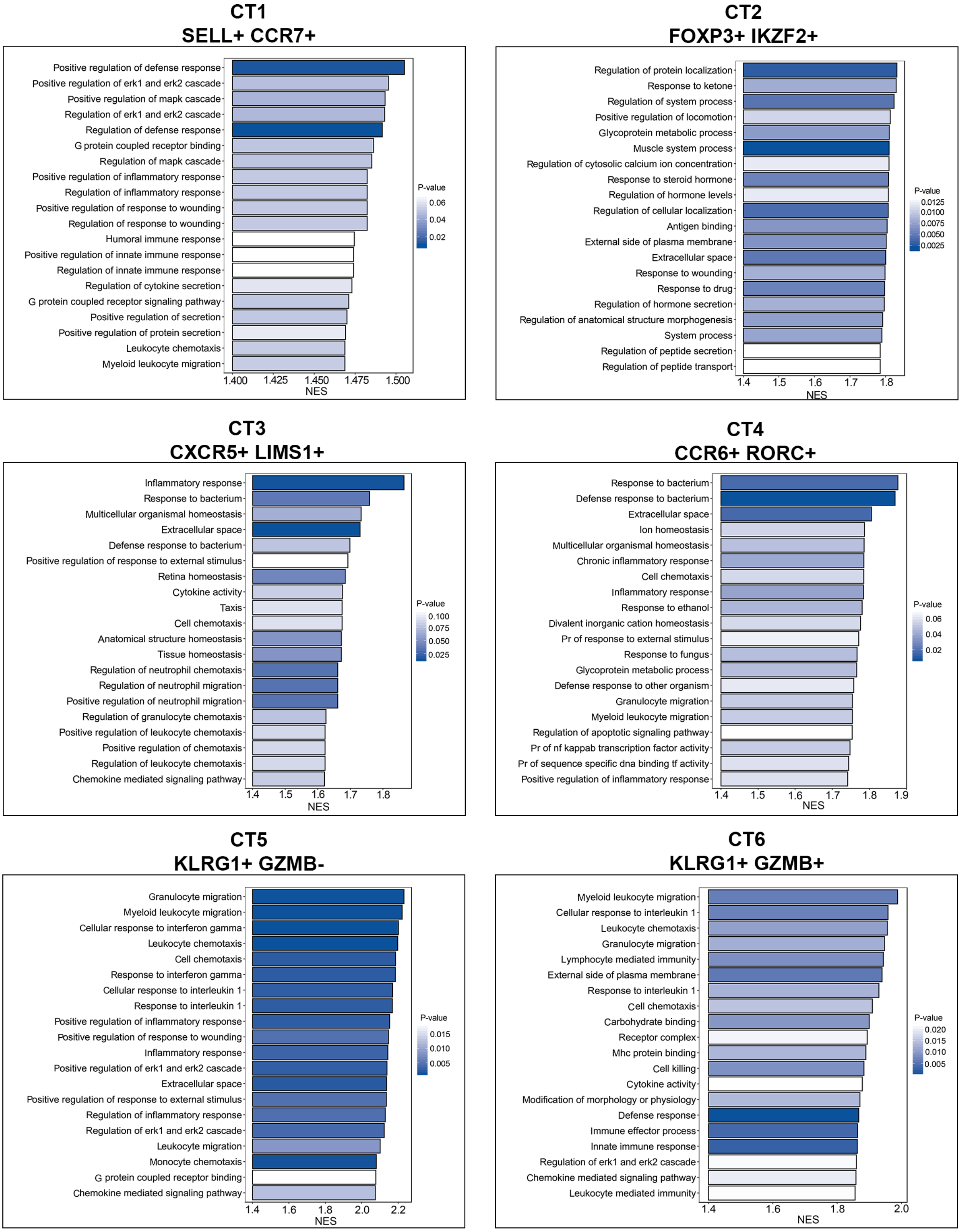

Supplementary Figure 8. The bar plot showed GSEA for the indicated gene sets (Gene Ontology terms) of six cell types between GO and GH. The pathways were ranked by NES, and top 20 pathways were shown. The gradient color represented P values. NES, normalized enrichment score.

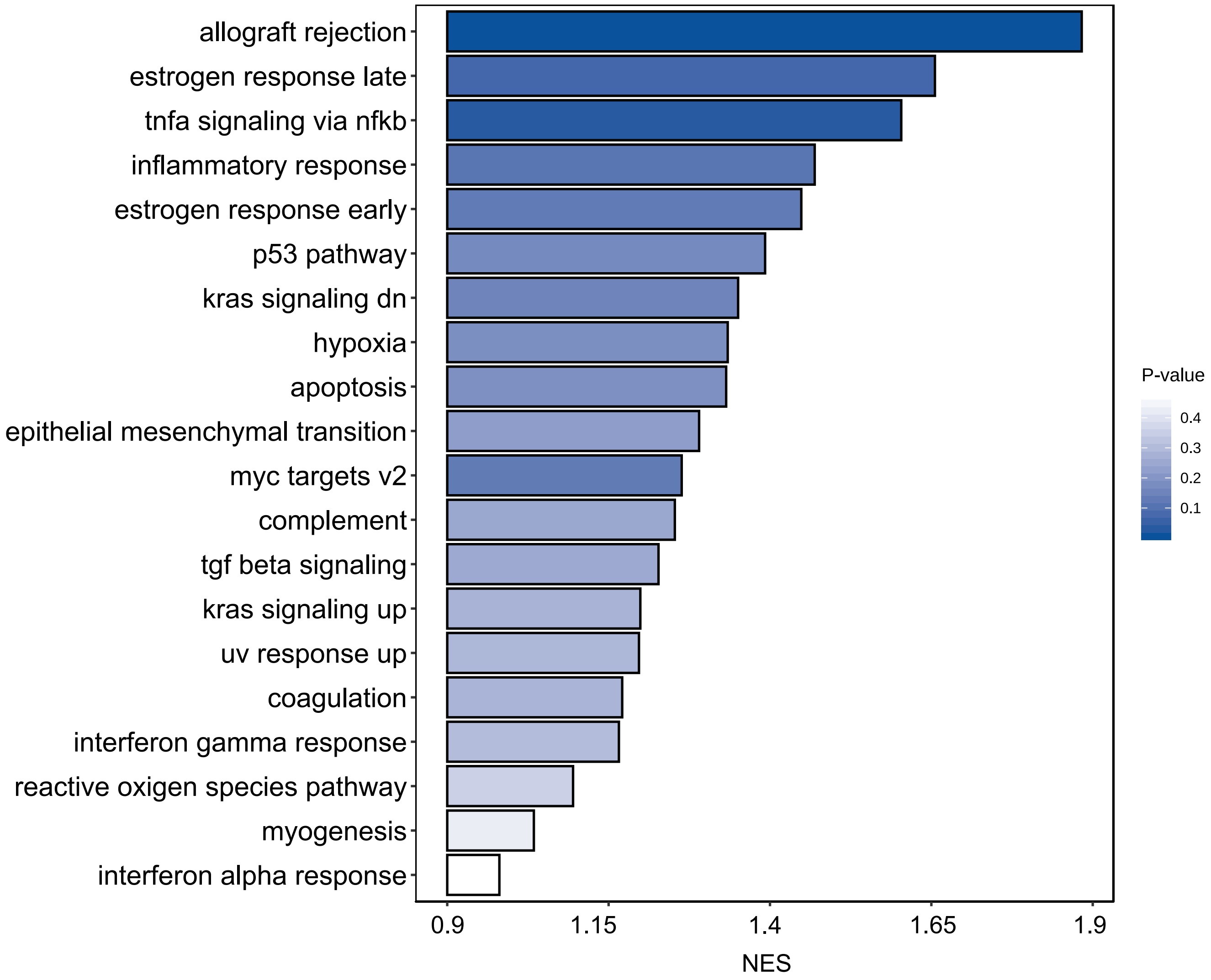

Supplementary Figure 9. GSEA analysis of hallmark gene sets in CT6 between GO and GH. The pathways were ranked by NES, and top 20 pathways were shown. The gradient color represented P values. NES, normalized enrichment score.

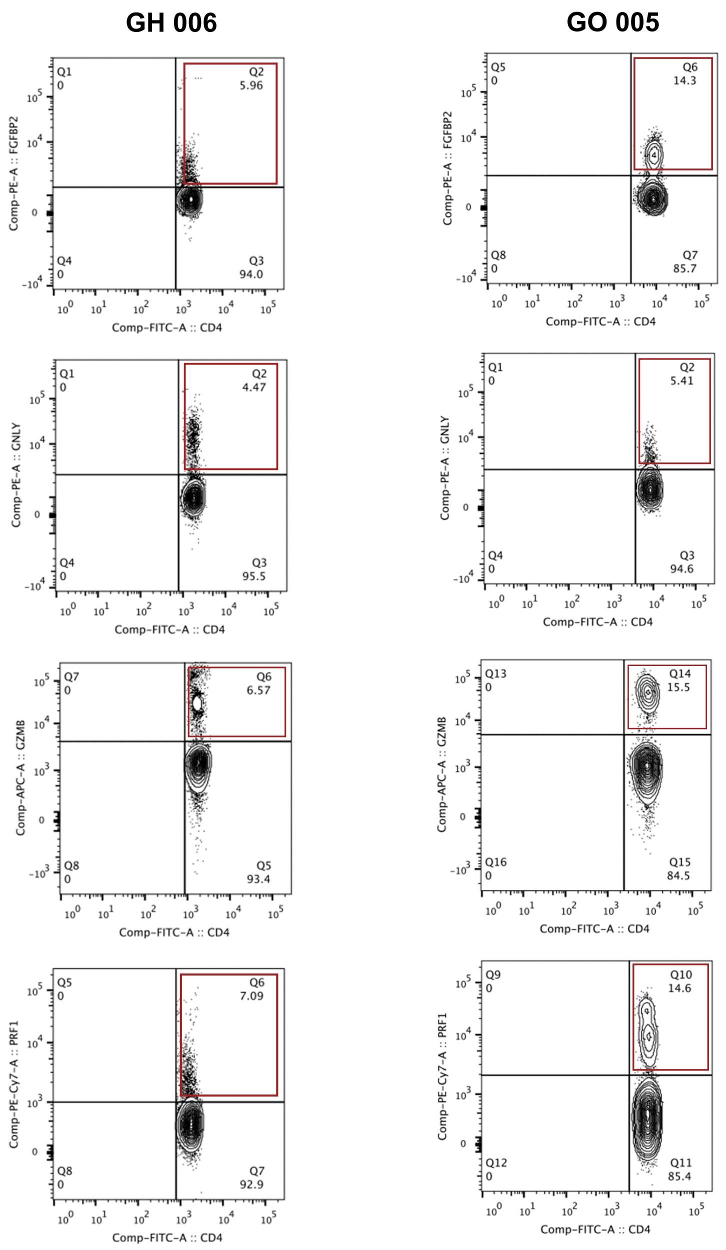

Supplementary Figure 10. Flow cytometry analysis showed the cytotoxic molecules in GO and GH. Flow cytometry analysis showed the surface expression of FGFBP2, and intracellular expression of GNLY, GZMB and PRF1 in singlet-gated CD4+ cells in representative GO and GH, respectively.

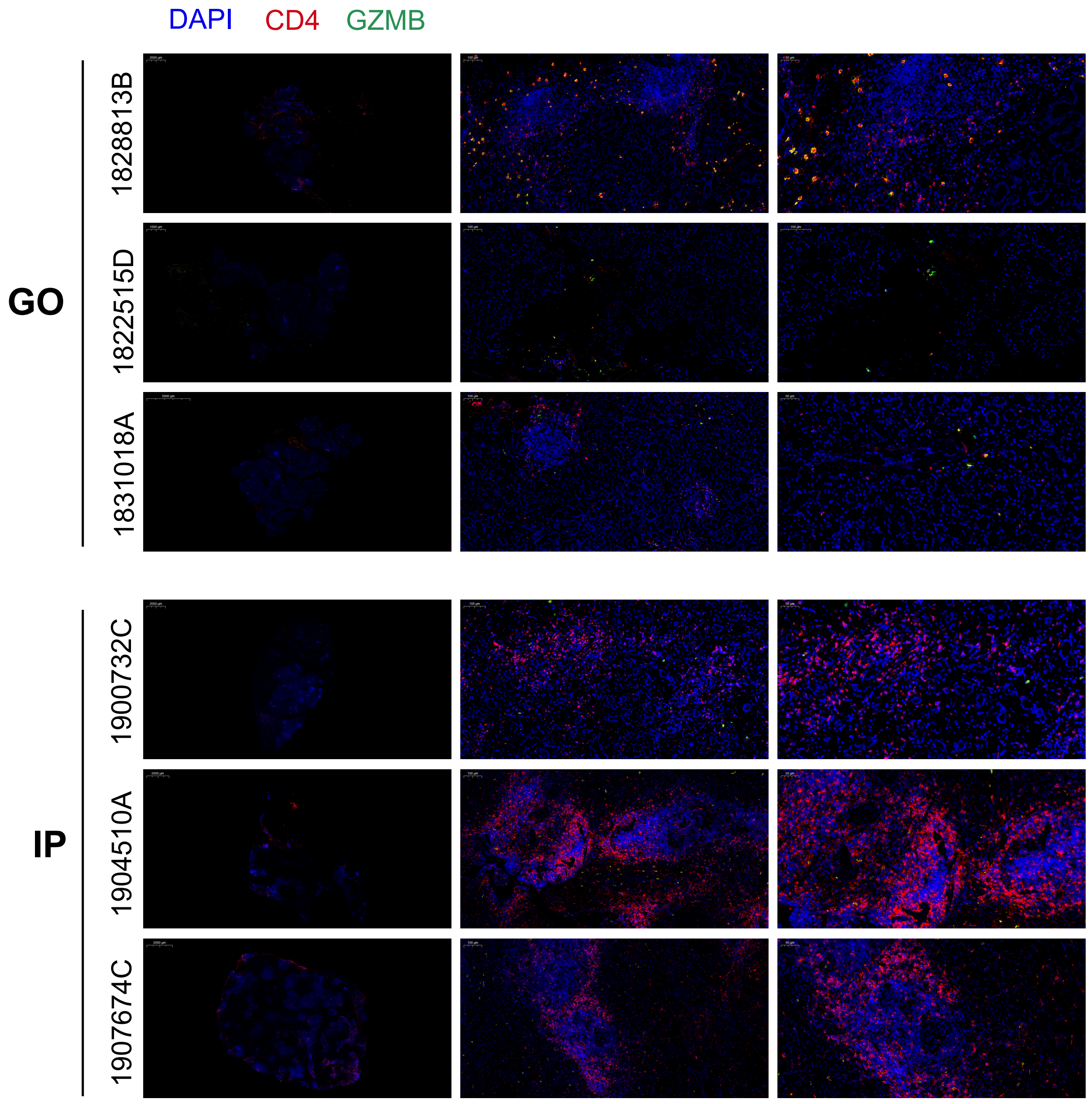

Supplementary Figure 11. Orbital tissues from GO and inflammatory pseudotumor (IP) patients by fluorescent multiplex immunohistochemistry showing coexpression of CD4 and GZMB at Low-magnification, 10× magnification and 20× magnification.

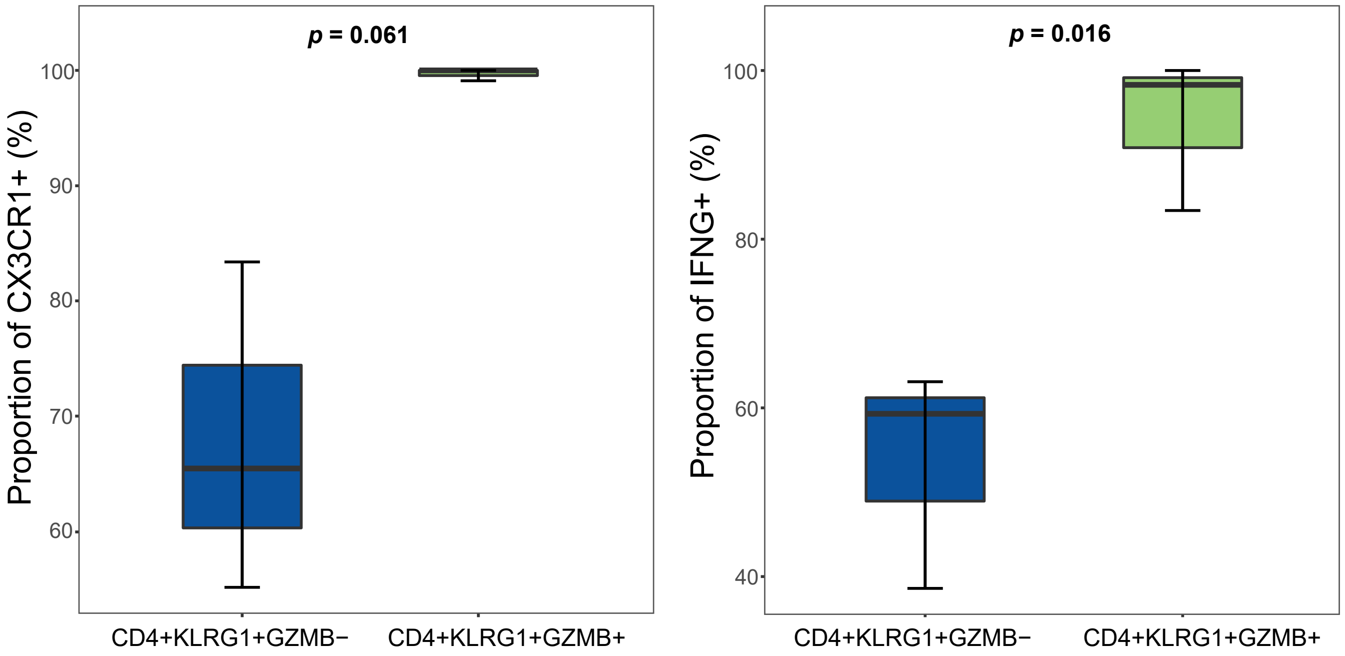

Supplementary Figure 12. Expression of chemotactic (CX3CR1) and inflammatory (IFNG) markers of CT5 (CD4+KLRG1+GZMB-) and CT6 (CD4+KLRG1+GZMB+) cells. Boxplots showed the proportion of CX3CR1 and IFNG in CD4+KLRG1+GZMB- and CD4+KLRG1+GZMB+ T cells derived from GO patients (n=3).

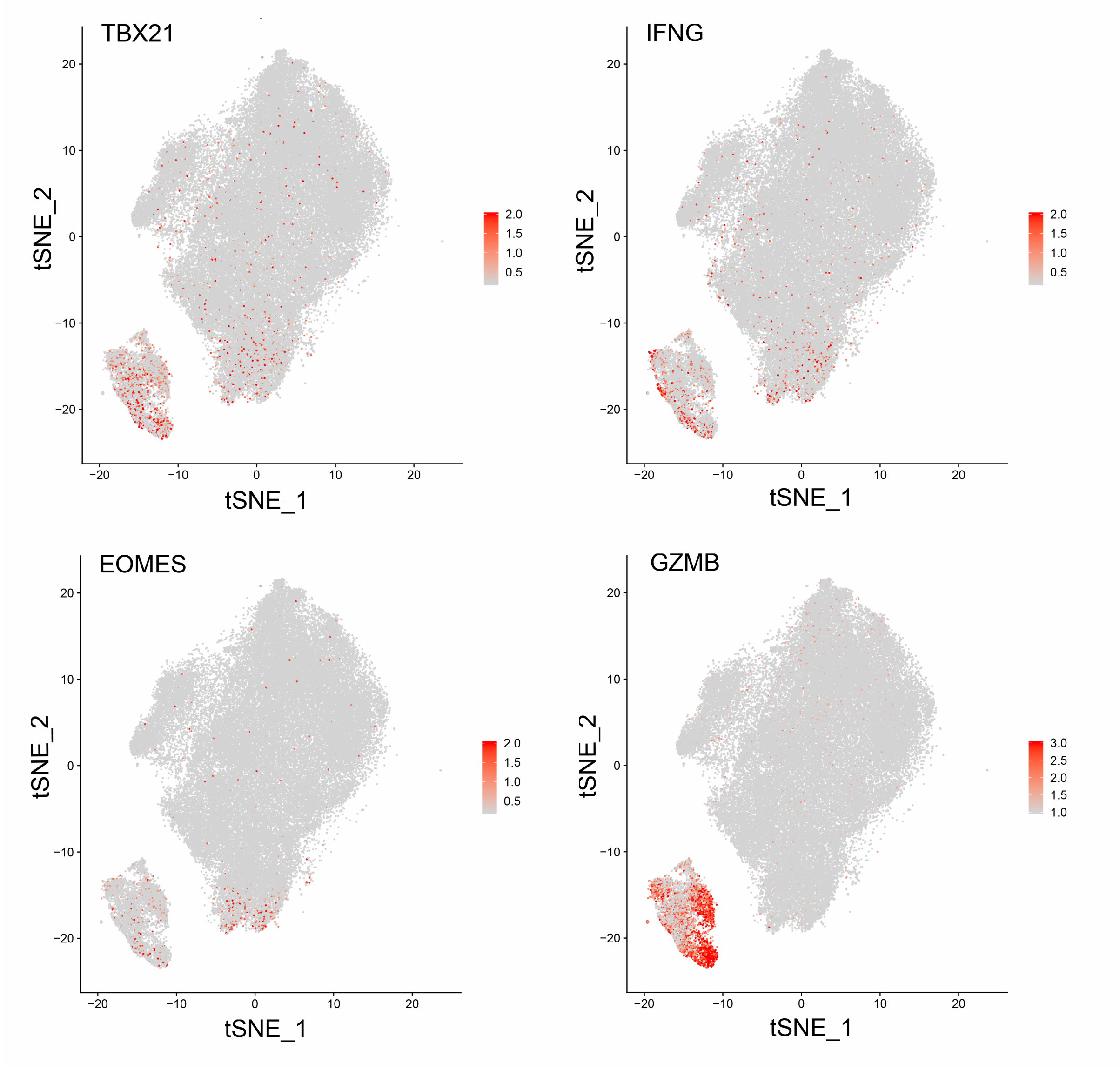

Supplementary Figure 13. Expression of canonical lineage markers of Th1 cells.

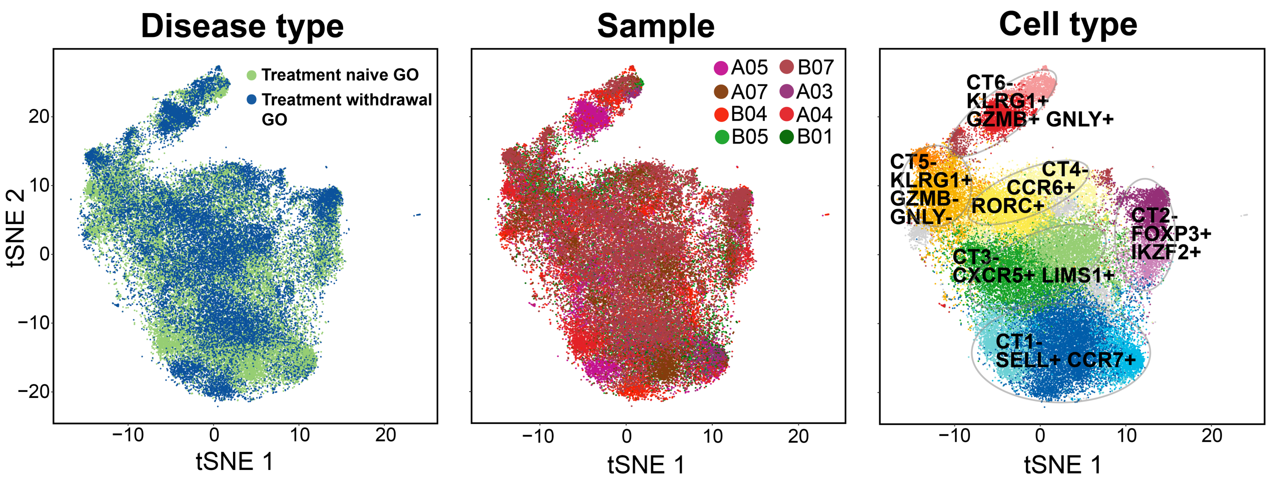

Supplementary Figure 14. Overview of 50,082 single CD4+ T cells from treatment naïve GO and treatment withdrawal GO patients. tSNE of cells profiling, with each cell colorcoded for (left to right): disease type (treatment naïve or treatment withdrawal), the corresponding patient and the cell types.

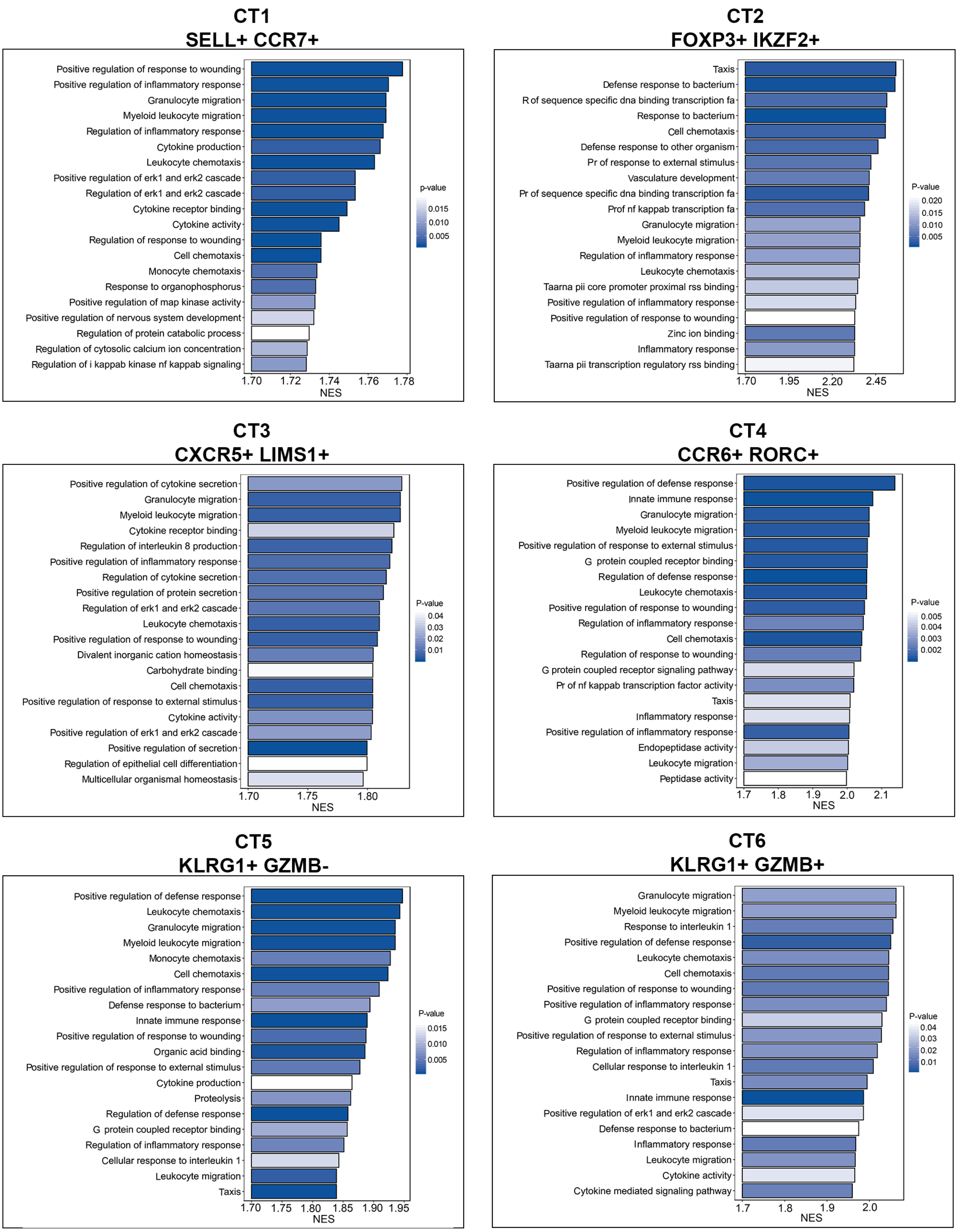

Supplementary Figure 15. GSEA of MP treatment effects on six cell types. The pathways were ranked by NES, and top 20 pathways were shown. The gradient color represented P values. NES, normalized enrichment score.

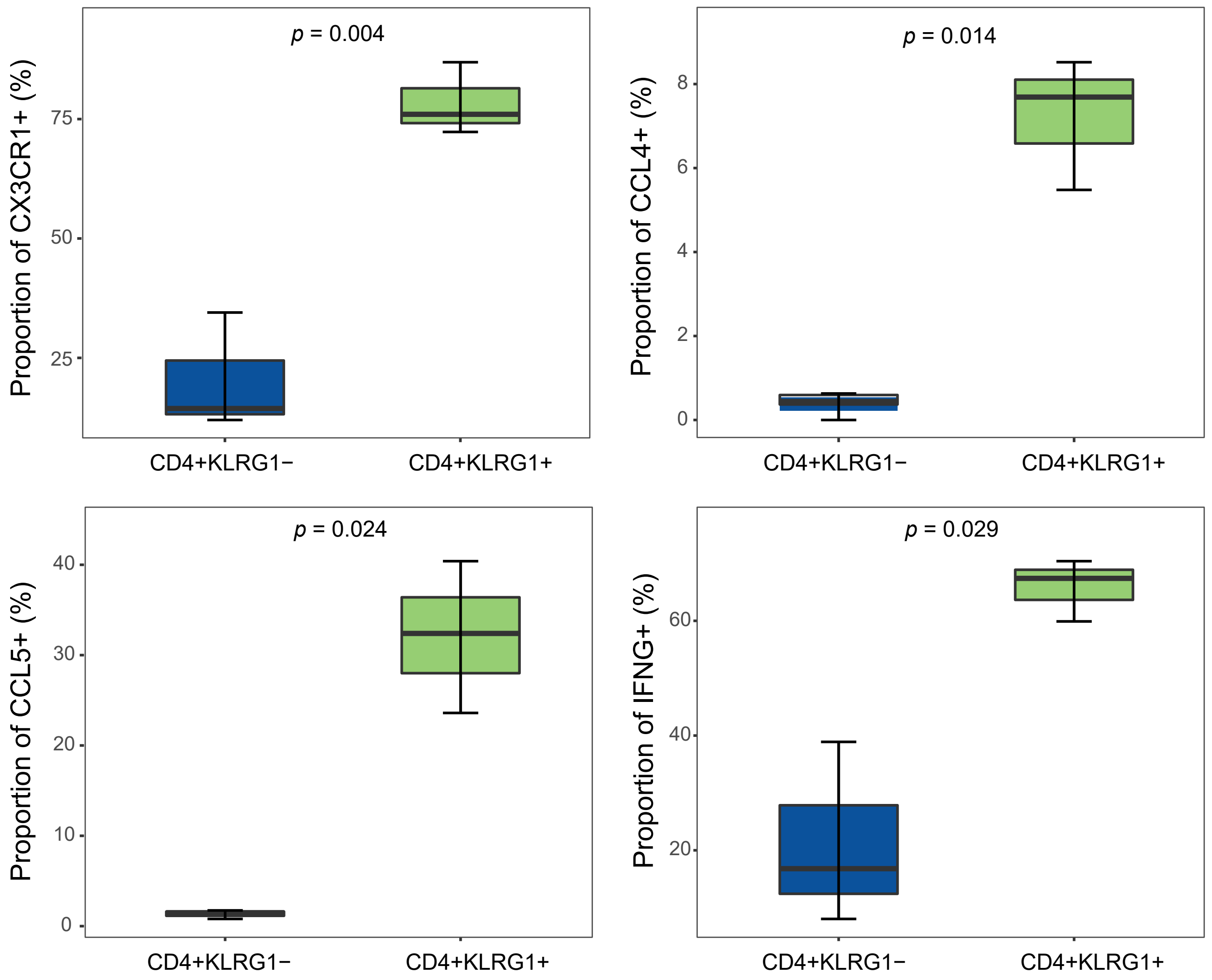

Supplementary Figure 16. Expression of chemotactic (CX3CR1, CCL4, CCL5) and inflammatory (IFNG) markers of CD4+KLRG1+ CTLs and CD4+KLRG1- T cells. Boxplots showed the proportion of CX3CR1, CCL4, CCL5 and IFNG in CD4+KLRG1+ CTLs and CD4+KLRG1- T cells derived from GO patients (n=3).
