# Supplementary material for: A novel CD4+ CTL subtype characterized by chemotaxis and inflammation is involved in the pathogenesis of Graves’ orbitopathy
Source: Cell Mol Immunol. 2021 Jan 29;18(3):735–45. doi: 10.1038/s41423-020-00615-2 (PMC8027210; doi:10.1038/s41423-020-00615-2)
Supplement: Supplementary file 2 — Supplementary Tables [file 41423_2020_615_MOESM2_ESM.pdf]

Supplementary Table 1 | Inclusion criteria and exclusion criteria for GO and GH patients in sc-RNA seq

GO	Inclusion criteria	1. Moderately to severely active GO;<br>2. Clinical Activity Score (CAS) ⩾ 3, CAS is based on the classical signs of acute inflammation (pain, redness, swelling, and impaired function) was proposed as a clinical classification to discriminate easily between the active and quiescent stages of the disease;<br>3. The grade of NOSPECS ⩾ 4. NOSPECS classification grades exclusively for clinical severity and does not provide a means of distinguishing inflammatory progressive. Class 4 refers to extraocular muscle involvement (usually with diplopia);<br>4. The duration of GO is less than 3 months;<br>5. The age in the range of [18, 65]
	exclusion criteria	1. Patients who were infected by fungus, mycobacterium tuberculosis, hepatitis virus and/or any other uncontrolled bacterium, virus and fungus;<br>2. Patients who are allergic to methylprednisolone;<br>3. Pregnant and lactating women;<br>4. Combined with other autoimmune diseases.<br>5. Patients who are suffering from active peptic ulcer, recent gastrointestinal surgery, serious diabetes, hypertension, hyperlipidemia, glaucoma, osteoporosis, heart disease, cardiac failure, hepatic impairment, impaired renal function and urolithiasis
GH	Inclusion criteria	1. Newly diagnosed (duration < 2 months);<br>2. The age in the range of [18, 65]
	exclusion criteria	1. Patients who were infected by fungus, mycobacterium tuberculosis, hepatitis virus and/or any other uncontrolled bacterium, virus and fungus;<br>2. Patients who are allergic to anti-thyroid drugs;<br>3. Pregnant and lactating women;<br>4. Combined with other autoimmune diseases;<br>5. Patients who are suffering from one or two manifestations, including pretibial myxedema, heart disease and hepatic impairment

Supplementary Table 2 | the category of data applied for analysis in this study

Analysis	Target cells	Samples
sc-RNA seq	CD4+ T	3 treatment naïve GH,<br>6 treatment naïve GO,<br>4 treatment withdrawal GO (the same patients with treatment naïve GO)
Flow cytometry	CD4+ T cell types identified by sc-RNA seq	26 GO, 9 GH, 10 HC
	Cytotoxic related molecules in GO and GH	
	CD4+KLRG1+ T cells and related cyctotoxic molecules	
Multicolor immunofluorescence	CD4, CD8, GZMB	5 GO (orbital tissue),<br>5 inflammatory pseudotumor (orbital tissue).
TCR-sequencing analysis	CD4+KLRG1+ T cells,<br>CD4+ naive T cells	7 GO
Degranulation assay and Multiple lymphocyte reaction	CD4+ T cells	8 GO

Supplementary Table 3 | Patient information and sequencing data for sc-RNA seq

Patient ID	Diagnosis	Gender	Age	FT4 (pmol/L)	CAS	Treatment	Target cell	Cell number	Mean reads per cell	Median number of genes per cell	Sequencing saturation
A02	GH	M	45	6.02	0	MMI	CD4+ T	7,342	74,451	1,502	84.9%
B02	GH	F	21	30	0	MMI	CD4+ T	11,221	51,419	1,278	82.4%
B03	GH	F	26	40.4	0	MMI	CD4+ T	6,490	68,193	1,329	84.3%
A06	GO	F	44	15.4	3	RAPA	CD4+ T	5,658	129,053	1,484	90.7%
B06	GO	M	56	14.3	6	IVMP+RAPA	CD4+ T	7,406	72,075	1,328	85.2%
B01	GO (B, A)	F	44	16.8	3	IVMP	CD4+ T	7,642	82,438	1,410	86.1%

B05				15.8	0	IVMP	CD4+ T	5,228	119,352	1,209	92.6%
A03	GO (B, A)	M	47	13.5	3	IVMP	CD4+ T	5,051	117,379	1,378	89.5%
A05				15.1	0	IVMP	CD4+ T	6,778	87,149	1,139	90.1%
A04	GO (B, A)	M	45	16.4	6	IVMP	CD4+ T	7,691	78,357	1,468	81.3%
B04				11.8	5	IVMP	CD4+ T	3,949	153,506	1,350	92.7%
A07	GO (B, A)	M	48	11.1	4	IVMP	CD4+ T	6,577	83,210	1,303	85.4%
B07				20.5	1	IVMP	CD4+ T	12,604	42,146	1,139	72.7%

B is before IVMP treatment. A is after IVMP treatment. M is male. F is female. FT4 is free thyroxine. CAS, Clinical Activity Score is based on the classical signs of acute inflammation (pain, redness, swelling, and impaired function) was proposed as a clinical classification to discriminate easily between the active and quiescent stages of the disease. MMI, methimazole; IVMP, pulses of intravenous methylprednisolone.

Supplementary Table 4 | Cell numbers and proportions of six cell types identified by sc-RNA seq

Cell type	Cell numbers	Proportion in CD4+ T cells
CT1- SELL+ CCR7+	22,322	37.33%
CT2- FOXP3+ IKZF2+	4,226	7.07%
CT3- CXCR5+ LIMS1+	10,347	17.30%
CT4- CCR6+ RORC+	8,212	13.73%
CT5- KLRG1+ GZMB-	5,969	9.98%
CT6- KLRG1+ GZMB+	6,041	10.10%
Unknown	2,678	4.48%
Total	59,795	100.00%

Supplementary Table 5 | Cell markers and percentages of flow cytometry for CD4+ T cells types identified by sc-RNA seq

Cell type	Cell markers (FACS)	Proportion in CD4+ T cells
CT1- SELL+ CCR7+	CD4+CD25-CD45RA-CCR7+	23.01% ± 5.34%
CT2- FOXP3+ IKZF2+	CD4+CD25+FOXP3+	1.98% ± 0.27%
CT3- CXCR5+ LIMS1+	CD4+CXCR5+ICOS+	6.18% ± 1.61%
CT4- CCR6+ RORC+	CD4+CCR6+CCR4+CXCR3-	2.85% ± 0.82%
CT5- KLRG1+ GZMB-	CD4+KLRG1+GZMB-	18.52% ± 9.85%
CT6- KLRG1+ GZMB+	CD4+KLRG1+GZMB+	7.39% ± 3.50%
